# The native microbiome of the nematode *Caenorhabditis elegans*: gateway to a new host-microbiome model

**DOI:** 10.1186/s12915-016-0258-1

**Published:** 2016-05-09

**Authors:** Philipp Dirksen, Sarah Arnaud Marsh, Ines Braker, Nele Heitland, Sophia Wagner, Rania Nakad, Sebastian Mader, Carola Petersen, Vienna Kowallik, Philip Rosenstiel, Marie-Anne Félix, Hinrich Schulenburg

**Affiliations:** Department of Evolutionary Ecology and Genetics, Zoological Institute, Christian-Albrechts University, Am Botanischen Garten 3-9, 24118 Kiel, Germany; Institute of Biology of the Ecole Normale Supérieure (IBENS), CNRS, Inserm, 46 rue d’Ulm, 75230 Paris Cedex 05, France; Institut Pasteur, 25-28 rue du Docteur Roux, 75724 Paris Cedex 15, France; Cologne Excellence Cluster for Cellular Stress Responses in Aging-Associated Diseases, University of Cologne, Joseph-Stelzmann-Str. 26, 50931 Cologne, Germany; Max Planck Institute for Evolutionary Biology, August-Thienemann-Strasse 2, 24306 Plön, Germany; Institute of Clinical Molecular Biology, Christian-Albrechts University, Am Botanischen Garten 1-3, 24118 Kiel, Germany

**Keywords:** *Caenorhabditis elegans*, Metaorganism, Microbiome, *Pseudomonas*, *Ochrobactrum*, *Stenotrophomonas*, *Sphingomonas*, Holobiont, Antifungal defense

## Abstract

**Background:**

Host-microbe associations underlie many key processes of host development, immunity, and life history. Yet, none of the current research on the central model species *Caenorhabditis elegans* considers the worm’s natural microbiome. Instead, almost all laboratories exclusively use the canonical strain N2 and derived mutants, maintained through routine bleach sterilization in monoxenic cultures with an *E. coli* strain as food. Here, we characterize for the first time the native microbiome of *C. elegans* and assess its influence on nematode life history characteristics.

**Results:**

Nematodes sampled directly from their native habitats carry a species-rich bacterial community, dominated by Proteobacteria such as Enterobacteriaceae and members of the genera *Pseudomonas*, *Stenotrophomonas*, *Ochrobactrum*, and *Sphingomonas*. The *C. elegans* microbiome is distinct from that of the worm’s natural environment and the congeneric species *C. remanei*. Exposure to a derived experimental microbiome revealed that bacterial composition is influenced by host developmental stage and genotype. These experiments also showed that the microbes enhance host fitness under standard and also stressful conditions (e.g., high temperature and either low or high osmolarity). Taking advantage of the nematode’s transparency, we further demonstrate that several Proteobacteria are able to enter the *C. elegans* gut and that an *Ochrobactrum* isolate even seems to be able to persist in the intestines under stressful conditions. Moreover, three *Pseudomonas* isolates produce an anti-fungal effect in vitro which we show can contribute to the worm’s defense against fungal pathogens in vivo.

**Conclusion:**

This first systematic analysis of the nematode’s native microbiome reveals a species-rich bacterial community to be associated with *C. elegans*, which is likely of central importance for our understanding of the worm’s biology. The information acquired and the microbial isolates now available for experimental work establishes *C. elegans* as a tractable model for the in-depth dissection of host-microbiome interactions.

**Electronic supplementary material:**

The online version of this article (doi:10.1186/s12915-016-0258-1) contains supplementary material, which is available to authorized users.

## Background

Bacteria have shaped the evolution of multicellular organisms since its very beginnings [1]. They are often essential for the survival and evolutionary fitness of animals, plants, and fungi and may even determine fundamental evolutionary events like speciation [2]. This interaction between multicellular hosts and their microbiome (that is the associated microbial communities that share their body spaces [3]) has only recently been recognized to be highly inter-dependent and the entire association, termed metaorganism [4] or holobiont [5], was thus proposed as a unit of natural selection [5, 6]. In particular, the associated microbiome can directly affect host development by providing food and essential metabolic compounds [7] or by acting as a stimulus for morphogenesis [8]. A healthy microbiome can also inhibit pathogen colonization by direct toxin-mediated interference [9], by limiting resources available to the invading microbes [10], or by directly modulating immune system maturation [11]. Conversely, a distorted microbiome can be cause of many diseases, for example, antibiotic-associated diarrhea caused by *Clostridium difficile* [12], obesity [13], or liver cirrhosis [14].

In spite of their potential importance, the microbiome of one of the most intensively studied model species, the nematode *Caenorhabditis elegans*, is currently unknown. Almost all work on this nematode is based on the canonical *C. elegans* strain N2, which has been adapted to laboratory conditions over decades [15], including the regular and routine removal of any microbes through hypochlorite treatment [16]. As a consequence, the N2 strain does not carry any microbes in its gut, on its surface, or anywhere else in its body (unpublished data, Félix and Schulenburg labs). In its laboratory environment, it is only confronted with its food bacterium, the *Escherichia coli* strain OP50. Microbiome associations are thus unknown to any of the numerous *C. elegans* researchers, who study the nematode under these conditions. In contrast, natural isolates of this taxon usually seem to contain a variety of microorganisms in their guts [17–19]. In general, worms are exposed to complex microbial communities in their preferred substrate in nature, namely decomposing plant material [17–19]. A more realistic and unbiased understanding of *C. elegans* biology urgently requires the explicit consideration of the worm’s natural microbiome [20–23]. A possible fitness benefit was already indicated upon gut colonization with certain non-pathogenic bacteria, leading to increased resistance against pathogens [24–26]. However, it is yet unclear whether any of the non-pathogenic bacteria used are associated with *C. elegans* isolates in nature.

The aim of the current study is to obtain first insights into the composition and function of the *C. elegans* microbiome. We characterized the microbial community of natural *C. elegans* isolates from North German locations collected in 2011 and 2012 [19, 27], and French and Portuguese samples collected between 2008 and 2013. The results were compared to the microbial communities found in the substrate samples, from which *C. elegans* was isolated, and also to those obtained from two congeneric species with similar habitat preferences, *C. briggsae* and *C. remanei* [19, 28]. To evaluate the influence of the microbiome on *C. elegans* life history, we isolated microbes from wild nematodes and used these to re-infect worms and thus to establish an experimental microbiome under laboratory conditions. Based on this approach, we assessed an influence of host developmental stage and genotype on bacterial community composition. We also tested the bacteria’s ability to colonize the worm gut, their effect on worm population growth under standard and stressful conditions, and also on fungal growth.

## Results and discussion

### *C. elegans* carries a distinct and species-rich microbiome

Our characterization of the native microbiome of *C. elegans* (See Methods for details) focused on samples from northern Germany [19, 27], France, and Portugal [19, 28]. For the German locations, we studied the congeneric *C. elegans* and *C. remanei* and their corresponding substrates (compost, rotting apples, vector invertebrates). For the remaining locations, we characterized isolates of *C. elegans*, *C. briggsae*, and *C. remanei*. Two types of worm samples were analyzed: (1) single worms isolated without any exposure to the standard laboratory food *E. coli* (denoted “natural worms”) and (2) worm populations grown on *E. coli* several weeks after isolation (denoted “lab-enriched worms”). In total, we studied 160 nematode and substrate samples from Germany, 20 worm samples from France, and one from Portugal (Additional file 1: Tables S1-1 and S1-2). Several measures were taken to minimize risk of contamination with airborne microorganisms and those from the nematode body surface, as explained in more detail in the Methods section. The bacterial microbiome was characterized for all samples using the V4 region of the 16S ribosomal DNA [29].

**Fig. 1:**
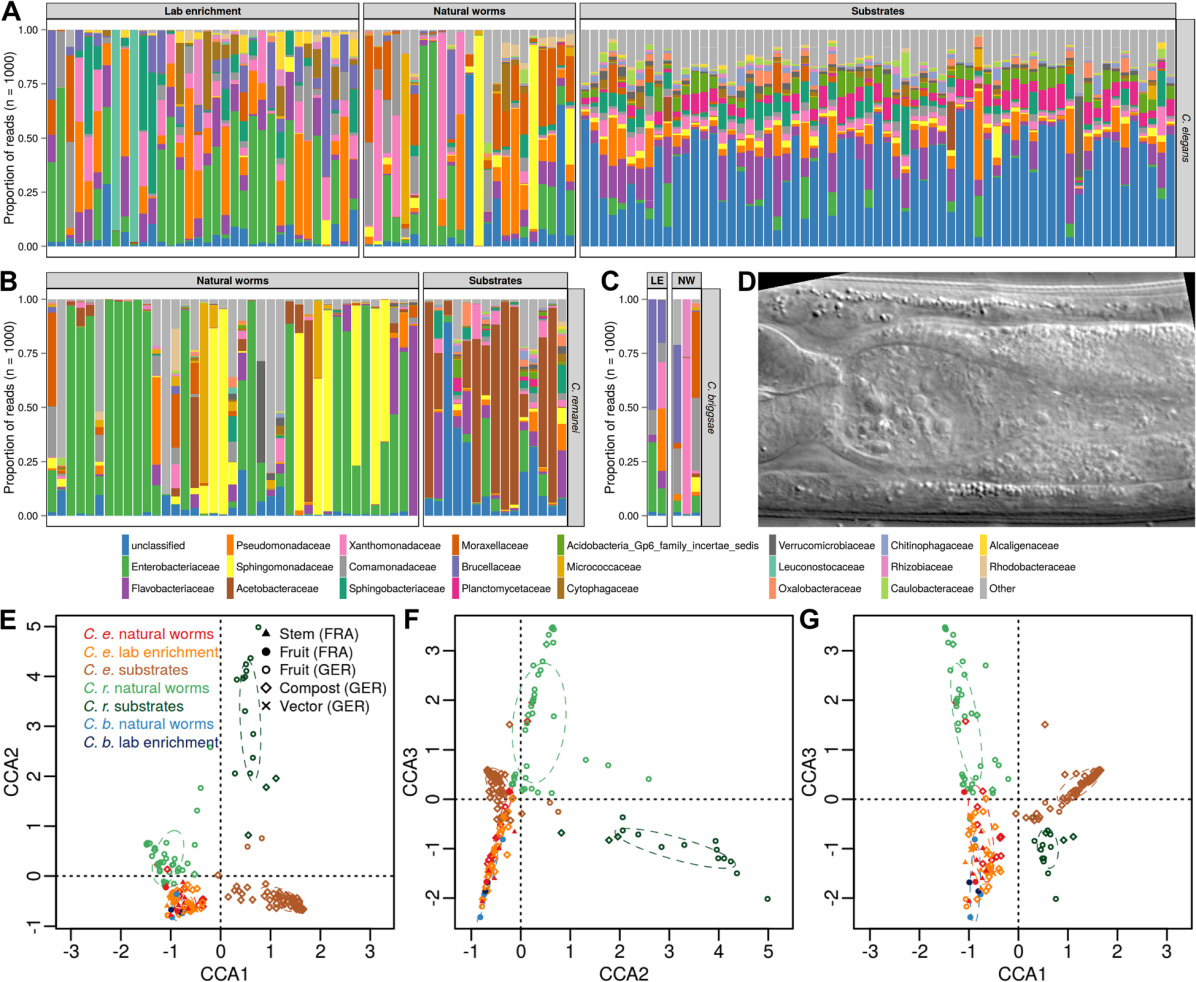
The native microbiome of the nematode *C. elegans*. **a**–**c** Frequency spectra of the bacterial classes based on MiSeq genotyping analysis for *C. elegans* (**a**), *C. remanei* (**b**), and *C. briggsae* (**c**), including results for natural worms (single worms), lab-enriched worms (nematode populations), and the corresponding substrates. **d** Differential interference contrast micrograph of *C. elegans* inhabited by its native microbiome. The anterior end of the worm is to the left. **e**–**g** Ordination by canonical correspondence analysis of bacterial operational taxonomic unit abundance in natural *Caenorhabditis* isolates and their substrates from German and French locations (indicated by colors and symbols; see legend), showing the three most significant axes. A three-dimensional visualization is given in Additional file 2. Statistics are provided in Additional file 1: Tables S1-6, S1-7, and S1-8. Detailed information about the samples is provided in Additional file 1: Tables S1-1 and S1-2. See also Additional file 3

We found the *C. elegans* microbiome to be rich in bacterial taxa (Fig. 1a–d). The most common operational taxonomic units (OTUs) included unclassified Enterobacteriaceae as well as members of the genera *Pseudomonas*, *Stenotrophomonas*, *Ochrobactrum*, and *Sphingomonas* (Additional file 1: Table S1-3). Individual samples from each nematode species differed substantially in their OTU compositions. At higher bacterial taxonomic order, however, samples from the same host species (either *C. elegans* or *C. remanei*) resembled each other and clearly differed to those from the corresponding substrates (Additional file 1: Tables S1-3, S1-4, S1-5 and S1-6; Fig. 1a–c).

Multivariate statistics (see Methods) supported the presence of a nematode-specific microbiome. Canonical correspondence analysis (CCA) identified a significant clustering of our samples by sample type (natural worms vs. lab-enriched worms vs. substrates; *P* ≤ 0.001; Additional file 1: Table S1-7) and species (*C. elegans*, *C. remanei*, and *C. briggsae*; *P* = 0.004; Additional file 1: Table S1-7). A post-hoc test revealed that samples from *C. elegans* and *C. briggsae* do not differ (*P* = 0.958), while both vary significantly from those of *C. remanei* (in both cases *P* ≤ 0.005). These differences are illustrated by the graphical ordination of the CCA (Fig. 1e–g, Additional file 2). The first axis separates all worms from the substrate samples, the second axis splits the *C. remanei* worms and substrate samples from all remaining material, while the third axis distinguishes the *C. remanei* worms from all remaining samples. All *C. elegans* samples and the few considered *C. briggsae* samples cluster closely together, irrespective of their location, substrate origin, or the worm isolation approach used (natural and lab-enriched worms). The CCA results are generally confirmed by permutational multivariate analysis of variance (ADONIS, [30, 31]) on Jaccard, Bray–Curtis, as well as unweighted and weighted UniFrac distances (Additional file 1: Table S1-8; Additional file 3).

Taken together, we demonstrate that *C. elegans* is associated with a species-rich microbiome dominated by Proteobacteria such as unclassified Enterobacteriaceae and the genera *Pseudomonas*, *Stenotrophomonas*, *Ochrobactrum*, and *Sphingomonas*. Previous microbial analyses of the *C. elegans* laboratory strain N2, after experimental exposure to rhizosphere [32] or compost-amended soil [26, 33], hinted at the possible importance of Proteobacteria. A recent study quantitatively assessed assembly of the microbial community in the N2 intestine, after worms were experimentally maintained in produce-enriched soil microcosms [34]. Although not all of the dominant microbial taxa are identical to those identified here (which could have resulted from differences in the experimental set-up and the nematode strains used), Berg et al. [34] similarly identified a high abundance of Enterobacteriaceae and Pseudomonadaceae, and then also Xanthomonadaceae and Sphingobacteriaceae. Members of these taxonomic groups may thus play a central role in the interaction with *C. elegans* and these bacteria can be easily retrieved from the environment; yet, their potential importance in the worm’s native microbiome is shown here for the first time by specific analysis of natural *C. elegans* isolates. The *C. elegans* microbiome is distinct from its substrate environment and from at least the congeneric *C. remanei*, while a possible difference to *C. briggsae* was difficult to assess due to small sample size. Our analysis additionally revealed that, even though the exact range of bacterial taxa showed variation among *C. elegans* samples, at higher taxonomic level the identified microbiome is stable across geographic distances and in the face of perturbations. This conclusion is supported by the overlapping microbiomes from natural and lab-enriched worms (Fig. 1e–g). The latter were isolated from their substrate on a plate with *E. coli* and subsequently cultivated with *E. coli* without any washing or sterilization steps. These worms were able to maintain the associated microbial community, even though they were propagated on agar plates in the presence of *E. coli*, strongly suggesting that the identified microbiome forms a relatively close relationship with these worms that is robust towards general environmental changes (natural environment vs. laboratory environment with *E. coli*). A stable species-specific microbiome is additionally supported by the concordance among *C. elegans* samples from different countries (Fig. 1e–g). These were independently collected by two different groups, using different isolation protocols and different substrate types. The nematode microbiome thus seems to show a similar species-specific signature, known from a wide variety of animals, ranging from the polyp *Hydra* [35] to primates [36].

### A *C. elegans*-specific experimental microbiome varies with developmental stage and genotype

As part of our 2011/12 sampling in Germany, we also obtained a total of 187 bacterial isolates, belonging to 29 bacterial genera from Proteobacteria, Bacteroidetes, Actinobacteria, and Firmicutes (Additional file 1: Table S1-9, Additional files 4 and 5). From these bacteria, we chose a subset of 14 isolates to establish an experimental microbiome. Nine of these were representative for the 15 most abundant identified genera of the *C. elegans*’ native microbiome, for which isolates were available (Additional file 1: Table S1-3). We added representatives of two other yet distinct abundant genera, namely *Comamonas* and *Rhodococcus*. We further included representatives of three additional genera, which belonged to abundant and taxonomically distinct bacterial orders of the worm’s native microbiome, such as *Achromobacter* (order Burkholderiales), *Bacillus* (order Bacillales), and *Microbacterium* (order Actinomycetales; Additional file 1: Table S1-3). The bacteria were provided on peptone-free agar, which minimizes bacterial proliferation, allowing experimental control of initial microbe frequencies. Sterilized nematode eggs were added and hatching worms raised until adulthood for three *C. elegans* genotypes: the laboratory strain N2 and two natural isolates, MY316 and MY379, from which most bacterial strains were isolated (Additional file 1: Table S1-9). DNA for microbial analysis was extracted from fourth instar larvae (L4), adult nematodes, and from the corresponding agar plates (See Methods and Additional file 1: Tables S1-10 and S1-11 for replicate numbers).

**Fig. 2:**
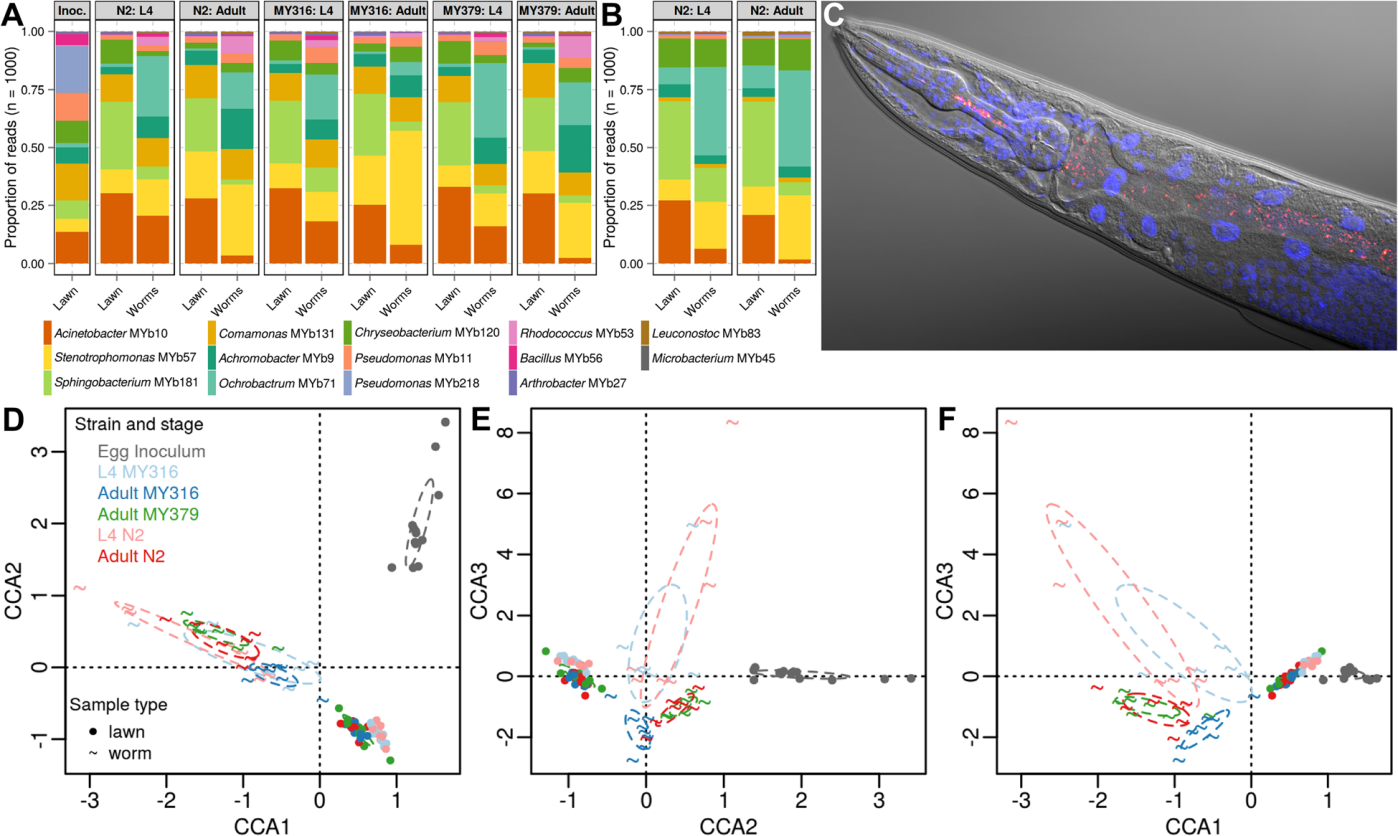
*C. elegans* cultured in the laboratory with a mix of 14 wild-caught bacteria retains a specific microbiome. **a**–**b** Average frequencies of bacterial taxa of the experimental microbiome for the lawns at different time points and two developmental stages (L4 and adult) of three *C. elegans* strains (N2, MY316, MY379; Additional file 1: Tables S1-12 and S1-15). **b** Shows the results for an independent experiment with N2 only. **c** Fluorescence micrograph of *C. elegans* inhabited by its experimental microbiome, visualized through fluorescence in situ hybridization of the bacteria with the general eubacterial probe EUB338 in red and DAPI staining of nematode cell nuclei in blue. The anterior end of the worm is to the left. See Additional file 6 for a three-dimensional illustration. **d**–**f** Canonical correspondence analysis of the experimental microbiome of *C. elegans*, showing the first three axes and including nematode stage (L4 or adult), sample type (nematode or lawn), and nematode strain (N2, MY316, or MY379) as factors (indicated by colors and symbols; Additional file 1: Tables S1-13, S1-14, and S1-16). A three-dimensional visualization is given in Additional file 8; see also Additional file 7 and Additional file 9. For all treatments, we considered at least six replicates. The only exception referred to the treatment combination worm-L4-MY379, for which only three replicates remained after quality control and which was thus excluded from further statistical analysis. Further details are given in Tables S1-10 and S1-11

Characterization of bacterial strain frequencies revealed a *C. elegans*-specific microbiome that is distinct from that on the agar plates (Fig. 2; Additional file 1: Table S1-12; Additional file 6). The most prominent example, the *Ochrobactrum* isolate MYb71, was found only in traces in the bacterial lawns, but consistently comprised 10–20 % of nematode bacterial communities, regardless of stage or strain. Other isolates specifically enriched in worms included *Achromobacter* MYb73, *Stenotrophomonas* MYb57, and to a lesser extent *Chryseobacterium* MYb7 and *Rhodococcus* MYb53 (Fig. 2a; Additional file 7).

Multivariate statistics such as CCA (Fig. 2d–f; Additional file 1: Table S1-13; Additional file 8) confirmed a significant influence of the sample type (*P* ≤ 0.001), the nematode stage (*P* ≤ 0.001), an interaction of stage and type (*P* ≤ 0.001), and also nematode strain (*P* = 0.037) on microbial community composition. These differences were also captured by the ADONIS assessment, where nematode and substrate samples formed distinct clusters, regardless of the used metric (Additional file 1: Table S1-14; Additional file 9). An independently performed experiment with the same 14 bacteria and the N2 nematode strain similarly demonstrated an effect of developmental stage on microbiome composition and increased quantities of *Ochrobactrum* MYb71 and *Stenotrophomonas* MYb57 in worms relative to agar plates (Fig. 2b; Additional file 1: Tables S1-15 and S1-16).

We conclude that *C. elegans* developmental stage and genotype can influence the exact composition of the nematode-associated bacterial community. Certain bacteria are enriched in the worm samples, especially *Ochrobactrum* MYb71 and *Stenotrophomonas* MYb57. These taxa may be part of *C. elegans*’ core microbiome.

### Some Proteobacteria can enter the nematode gut under experimental conditions

**Fig. 3:**
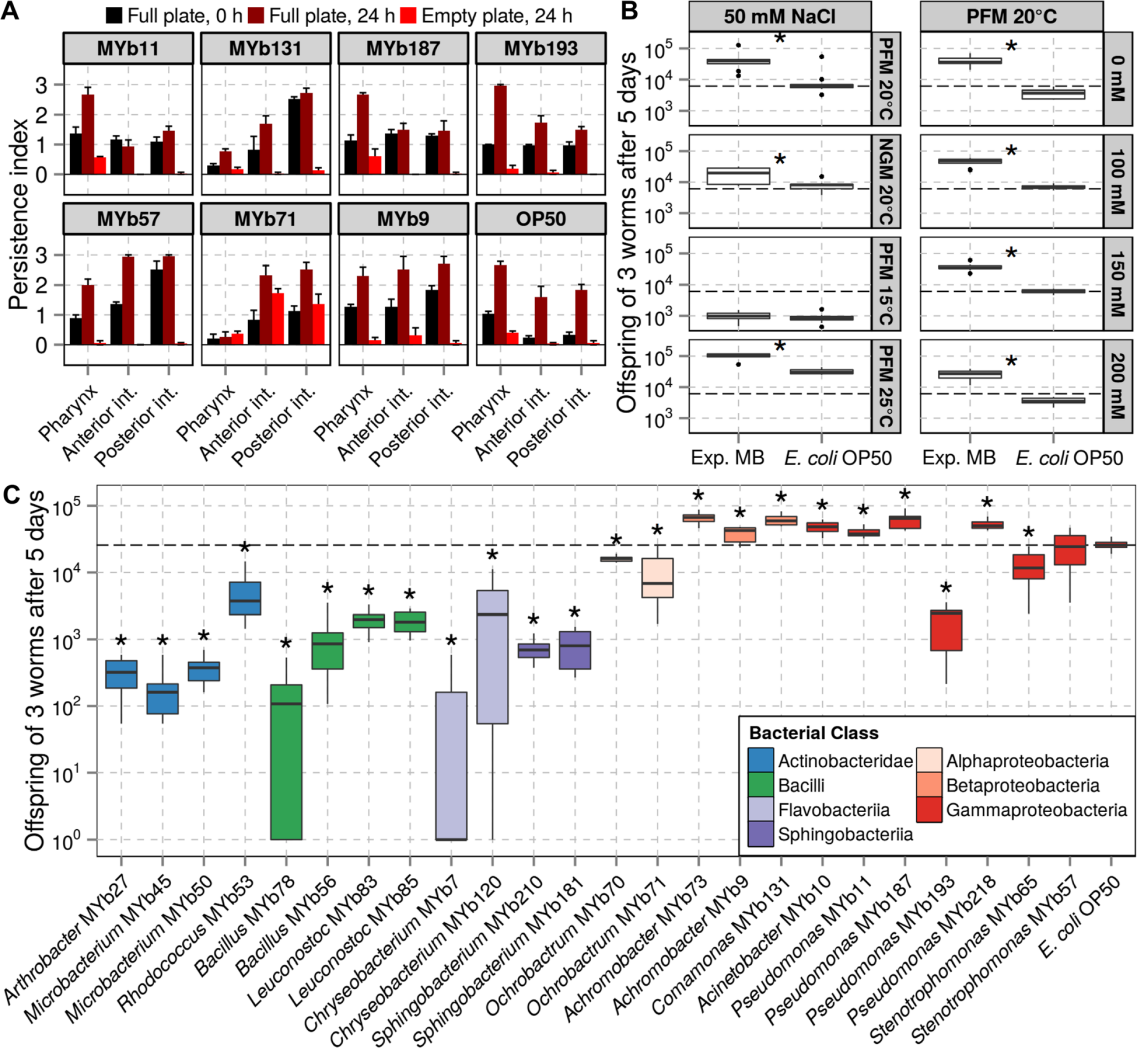
Interaction of individual bacterial isolates with *C. elegans*. **a** Persistence of bacterial isolates in the *C. elegans* gastrointestinal tract. We assessed the presence of selected bacteria at the beginning of the experiment (0 h, black color) and after 24 h on either a lawn of the same bacterium (dark red colour) or an empty plate (light red colour), using fluorescence in situ hybridization with eubacterial probe EUB338. Bacterial load was quantified in four categories: (0) absent, (1) single cells, countable, (2) clumps of cells, too many to count, and (3) region is filled. Results are shown for an *E. coli* control and seven bacterial isolates: *Pseudomonas* MY11b, *Comamonas* MY131b, *Pseudomonas* MY187b, *Pseudomonas* MY193b, *Stenotrophomonas* MY57b, *Ochrobactrum* MY71b, and *Achromobacter* MY9b. Barplots show the mean bacterial load of 10 worms with standard error of the mean of a total of three independent replicates (raw data in Additional file 1: Table S1-17). **b**, **c** Nematode population size on either the experimental microbiome community (**b**) or individual bacteria (**c**). Population size was measured as total offspring of three N2 hermaphrodites after 5 days. In (**b**), population size was determined under standard and different stress conditions, including normal nematode growth medium (NGM), peptone-free medium (PFM) at three temperatures (15 °C, 20 °C, 25 °C), and five salt concentrations (0–200 mM NaCl). Please note that the standard laboratory growth conditions for *C. elegans* in our lab consist of either PFM or NGM at 20 °C and 50 mM NaCl. In (**c**), all experiments were performed on PFM at 20 °C and 50 mM NaCl. The dashed lines indicate the median worm fitness on *E. coli* OP50 under the respective control conditions of the respective experiments. Asterisks denote significant differences from the *E. coli* control (*, α ≤ 0.05, false discovery rate-corrected for (c), Additional file 1: Tables S1-19 and S1-21, *n* ≥ 10; the raw data is provided in Additional file 1: Tables S1-18 and S1-20). Colors highlight the different bacterial groups

We assessed the ability of individual Proteobacteria to enter the nematode gut by using microscopic analysis, taking advantage of the worms’ transparency (Methods). Nematodes were raised on single bacterial isolates until adulthood and then transferred to new plates containing either the same or no bacterium, always using peptone-free nematode growth medium (NGM) agar plates. Bacterial abundance was studied at 0 and 24 h post transfer, using fluorescence in situ hybridization (FISH; Additional file 6; Additional file 1: Table S1-17). At 0 h (directly before transfer), bacteria were present in all parts of the gut; 24 h after transfer, most bacterial isolates remained abundant, especially *Stenotrophomonas* MYb57, *Achromobacter* MYb9, and *Ochrobactrum* MYb71, which reached higher frequencies than the standard laboratory food *E. coli* OP50 (Fig. 3a). Intriguingly, the *Ochrobactrum* MYb71 isolate also remained at high frequency inside the nematode gut when these were maintained without any bacterial lawn (red bars in the MYb71 panel, Fig. 3a). This was not the case for any of the other tested bacteria.

We conclude that several bacterial isolates are able to enter and most likely colonize the nematode gut, consistent with the above results from the experimental microbiome (Fig. 2a, b). As the nutrient-lacking peptone-free medium (PFM) does not support bacterial growth and as some isolates appeared to reach higher abundances than *E. coli*, these bacteria are unlikely to serve exclusively as food but may be able themselves to use the nematode gut as an environmental niche. Moreover, at least one of the isolates, *Ochrobactrum* MYb71, seems to be able to persist in the nematode gut under stressful conditions of an empty plate. Under these conditions, the bacteria should have been used by the worms as food and/or eliminated by general stress responses, which can be induced under these conditions and can include upregulation of immune effector genes such as lysozymes [37, 38]. This observation suggests that the *Ochrobactrum* isolate is capable of long-term interactions with the nematode. Bacteria of the genus *Ochrobactrum* are common in soil [39, 40] and found in various animals ranging from humans [41] to invertebrates, including nematodes [32, 39, 42, 43]. In our case, the same *Ochrobactrum* MYb71 isolate was among the most prevalent bacteria in the experimental worm microbiome, while being almost undetectable on the corresponding agar plates (Fig. 2a, b). *Ochrobactrum* is also one of the common taxa in the nematode’s native microbiome (Fig. 1a; Additional file 1: Table S1-3) and was indicated in a previous study to coexist with *C. elegans* in nature [43]. These findings may suggest that *Ochrobactrum* uses *C. elegans* as a growth niche, apparently without causing major fitness reductions (see below). This stands in strong contrast to previous reports of gut-persisting bacteria in *C. elegans*, such as *Salmonella* Typhimurium [44], *Serratia marcescens* [45], and *Enterococcus* spp. [46], which are all pathogenic. It is currently unclear how exactly this bacterium may obtain energetic resources inside the host without producing major harm. This could be achieved by using host waste products available in the gut and/or by stimulating and retrieving specific compounds, which can be produced by the host without major energy requirements.

In case of the other tested bacteria, their failure to persist in the experimental monoinfections was surprising. Many of the same taxa persisted in the lab-enriched worms (colonized in nature) although these were cultured up to 3 weeks on peptone-free agar plates with the laboratory food *E. coli* (Fig. 1). These discrepancies may be explained by the specific laboratory conditions used during the reinfection experiments, which were likely stressful for worms because of the absence of food, and/or by additional bacteria-bacteria interactions possible in the experimental microbiome but not the monoinfections.

### The experimental microbiome and individual bacterial isolates enhance *C. elegans* population growth

We next asked whether our experimental microbiome influences nematode population growth, a proxy for evolutionary fitness. We initiated worm cultures with three L4 on the mixture of 14 bacterial isolates. Population size was measured after 5 days under standard laboratory and various stress conditions, including the normal NGM, the PFM at three different temperatures (15 °C, 20 °C, 25 °C), and also the PFM at five salt concentrations (0–200 mM NaCl; Methods). Our analysis revealed that the experimental microbiome significantly enhances nematode population growth relative to the *E. coli* control under all conditions (*P* < 0.05; Fig. 3b; Additional file 1: Tables S1-18 and S1-19) except of the experiment at 15 °C (*P =* 0.31).

We subsequently tested 24 individual bacterial isolates under standard conditions (PFM with 50 mM NaCl at 20 °C). These 24 isolates included the same 14 isolates used for the experimental microbiome. We considered 10 additional isolates from the same genera (Additional file 1: Tables S1-3 and S1-9) in order to obtain a first indication of within-genus variation of the bacteria’s interaction with *C. elegans*. In these experiments, almost all Proteobacteria led to significantly higher population growth than *E. coli. Achromobacter* MYb73, *Comamonas* MYb131, and *Pseudomonas* MYb187 even caused a more than 2.5-fold increase in offspring production (*P* ≤ 0.01; Fig. 3c; Additional file 1: Tables S1-20 and S1-21). Most remaining Proteobacteria supported population sizes only slightly, albeit significantly, smaller than the *E. coli* reference. The tested members of the Actinobacteridae, Bacilli, Flavobacteriia, and Sphingobacteriia all significantly reduced population size.

In conclusion, the experimental microbiome enhances population growth relative to *E. coli* under the tested conditions, including stressful environments of high temperature and either low or high osmolarity. The comparison between results with the microbiome mixture and the single isolates under standard conditions suggests that a fitness increase can be caused by members of the Proteobacteria, including taxa such as *Achromobacter*. Other Proteobacteria specifically enriched in nematodes (e.g., *Stenotrophomonas* and *Ochrobactrum*) support similar or slightly reduced population growth relative to the *E. coli* control, suggesting neither growth-promoting nor pathogenic effects under these conditions. In several cases, we observed contrasting effects among bacterial isolates of the same genus. These differences might arise from intra-taxon variation in the bacteria’s ability to produce nutritious substances and/or harmful compounds for the nematodes. Some variation in the effect on nematode growth rate was previously shown for soil bacteria [26, 47–50], although not including bacteria obtained from natural *C. elegans*, as in the present study.

### Members of the *C. elegans* microbiome exhibit anti-fungal activity

**Fig. 4:**
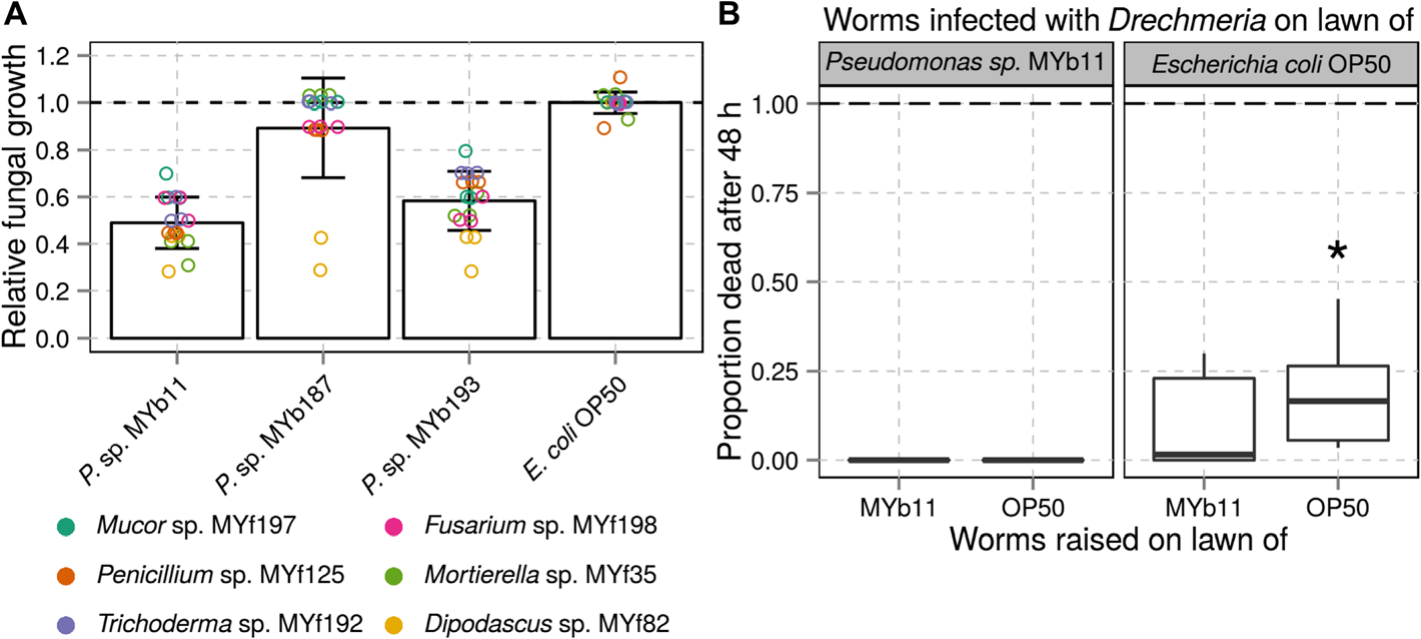
*Pseudomonas* isolates from the *C. elegans* microbiome inhibit fungal growth. **a** In vitro effect of three *Pseudomonas* isolates (relative to *E. coli* OP50) on growth of six selected fungi, isolated from natural *Caenorhabditis* samples, after 3 days (*n* = 3, statistics in Table S1-23 and raw data in Additional file 1: Table S1-22). Each fungus is indicated by a different colour. **b** Effect of the *Pseudomonas* isolate MY11b on *C. elegans* mortality induced by the fungal pathogen *Drechmeria coniospora* (*n* = 6, statistics in Table S1-25 and raw data in Additional file 1: Table S1-24). Worms were either grown on *Pseudomonas* MY11b or *E. coli* (indicated on X axis) and then exposed to the fungal pathogen either in the presence of *Pseudomonas* MY11b or *E. coli* (given by the two panels)

An intact bacterial microbiome can be an effective barrier against fungal infections [51]. *Pseudomonas* spp. are well-known for their anti-fungal secondary metabolites [52]. Therefore, we tested three *Pseudomonas* isolates (MYb11, MYb187, MYb193) for their activity against six fungi, similarly obtained from natural *C. elegans* (Additional file 1: Table S1-9; Additional file 4B). All three *Pseudomonas* impaired fungal growth in comparison to *E. coli*, although with varying efficiencies (Fig. 4a; Additional file 1: Tables S1-22 and S1-23). MYb11 and MYb193 produced broad anti-fungal activity, significantly reducing the growth of all six fungi (*P* ≤ 0.001), while MYb187 caused significant growth inhibition of only one fungal isolate, *Dipodascus* MYf82 (*P* ≤ 0.001).

We next asked whether the antifungal effect of one of the *Pseudomonas* isolates, MYb11, also benefitted *C. elegans* confronted with its fungal pathogen *Drechmeria coniospora* [53]. Worm populations were raised on either the *Pseudomonas* isolate MYb11 or *E. coli* and then as adults exposed to the fungus in the presence of either *Pseudomonas* MYb11 or *E. coli* in a full factorial design. The presence of *Pseudomonas* MYb11 during pathogen exposure completely prevented nematode death after 48 h, irrespective of the bacteria present during worm development (Fig. 4b; Additional file 1: Tables S1-24 and S1-25). Moreover, when worms were raised on *Pseudomonas* MYb11 and then exposed to the fungal pathogen in the presence of *E. coli*, they still suffered substantially less mortality than the corresponding worm populations raised on *E. coli* (Fig. 4b; Additional file 1: Table S1-25)*.*

In summary, we identified members of *C. elegans*’ microbiome with an antifungal effect that can protect the nematode from fungal pathogens in vivo. Protection seems to be provided by an antifungal compound produced by the bacteria (Fig. 4a, b, left panel) and, most likely, by a host factor induced while the nematode is exposed to the bacterium during development (Fig. 4b, right panel). Similar protective, anti-pathogenic effects in *C. elegans* were previously demonstrated for other bacteria such as *Lactobacillus acidophilus* [25] and *Pseudomonas mendocina* [26]. Our study is the first to suggest this for members of the native *C. elegans* microbiome.

## Conclusions

We present the first systematic analysis of the native microbiome of the model organism *C. elegans*, which is characterized by Proteobacteria including *Ochrobactrum*, *Pseudomonas*, *Stenotrophomonas*, and *Sphingomonas*. We anticipate that future consideration of the microbiome will improve our functional understanding of *C. elegans* signaling processes and genetic mechanisms and enhance discovery of novel functions for the, at least, 40 % of nematode genes without current annotation [23]. Our work lays the necessary groundwork for *C. elegans* to become an experimental model for native microbiome research allowing utilization of the genetic tractability of the worm to address pressing questions about microbiome-determined host biology.

## Methods

### Sampling locations and nematode lines

In northern Germany, we collected *C. elegans* and *C. remanei* nematodes from four sampling sites in 2011 and 2012 (Additional file 1: Table S1-1). A detailed description of the sampling sites and substrates has been previously published [19]. Experiments were performed using the laboratory strain N2 or the northern German *C. elegans* wild isolates MY316 and MY379, following standard maintenance procedures [16]. *C. elegans*, *C. briggsae*, and *C. remanei* were also isolated from nine locations in central and northern France and in one case Lisbon, Portugal (Additional file 1: Table S1-1; [19, 28]).

### Isolation, characterization, and maintenance of associated microbes

Microbial isolates were obtained from the northern German samples, either using aliquots of frozen worm populations or from environmental substrates, from which we successfully isolated *C. elegans* (Additional file 1: Tables S1-1 and S1-2). For microbe isolation, the respective nematode samples were washed three times in 1 mL M9, then resuspended in 250 μL M9 containing three 1-mm zirconium beads, followed by 2 min vortexing to break up the worms. The environmental samples were taken up in sterile ddH_2_O and shaken for 1 h to suspend the microbes present. Afterwards, the solid particles in both sample types were pelleted by centrifugation and 100 μL of supernatant in serial dilutions were plated onto 9-cm agar plates containing either diluted trypticase soy agar (TSA, 10 % strength), MacConkey agar, Sabouraud glucose agar, potato dextrose agar, or yeast peptone dextrose agar. Culture plates were incubated at 15 °C, to simulate average temperature conditions in the northern German sites. Single colonies were picked upon appearance and re-cultured for purification on diluted TSA. For long-term storage, stocks with 30 % glycerol were prepared from fresh liquid cultures in trypticase soy broth (TSB) and stored at −80 °C.

The microbial material was used to select specific isolates as representatives of the abundant genera from the native *C. elegans* microbiome, as inferred from bacterial MiSeq genotyping analysis (Fig. 1 and Additional file 1: Table S1-3). For bacteria, taxonomic identity was determined through Sanger-sequencing of the complete 16S rRNA gene using the primers 27f (AGAGTTTGATCMTGGCTCAG) and 1492r (AAGTCGTAACAAGGTAACC) [54], as well as 701f (GTGTAGCGGTGAAATGCG) and 785r (GGATTAGATACCCTGGTAGTCC). Fungal taxonomic identity was inferred through sequencing of the ribosomal internal transcribed spacer using primers ITS1f (CTTGGTCATTTAGAGGAAGTAA) [55] and ITS4r (TCCTCCGCTTATTGATATGC) [56]. The approximate taxonomic position of the isolates was subsequently assessed with the help of a BLAST-based similarity analysis, which is sufficient for an approximate classification of the isolates, especially at higher taxonomic levels and as required at this particular step, even though exact species designations may not always be correct. In particular, the sequences were aligned to obtain a single consensus sequence per isolate and these consensus sequences were compared to NCBI’s nucleotide data base using NCBI BLAST [57].

In addition, we used a phylogeny-based approach for a more reliable taxonomic classification of the 24 bacterial and six fungal isolates, which were characterized in more detail at the phenotypic level. We performed six separate phylogenetic analyses: one for the considered fungi and one each for the Actinobacteria, the Firmicutes, the Alpha-/Beta-Proteobacteria, and also the Gamma-Proteobacteria. For each of these groups, we compared DNA sequences of our isolates to either bacterial 16S type strain sequences obtained from the Ribosomal Database Project (RDP, [58]) or fungal ITS sequences, comprising both reference and representative sequences, obtained from UNITE [59]. Several of the considered genera had a large number of type sequences in the database (e.g. RDP has 140 type strain sequences of the genus *Pseudomonas* alone). To enhance efficiency of the analyses, we chose the most similar sequences (10 for bacteria, five for fungi) to our isolates by BLASTing the latter against the respective type sequences. For each of the six groups, we subsequently created multiple sequence alignments with the program MUSCLE [60] using the sequences obtained by us for our isolates, the chosen reference type sequences, and also always several outgroup taxa, either from the same bacterial phylum (all bacterial analyses) or from the major fungal clades (the fungal analysis). Phylogenetic inference was based on Maximum Likelihood. The optimal substitution model was identified using JModelTest2 [61]. Maximum-likelihood based tree reconstruction and parameter optimization was performed using PhyML [62]. Trees were visualized by the R-package *ape* [63] and shown in Additional file 5.

Prior to the phenotypic assays, bacteria and fungi were streaked out on TSA plates and incubated at 25 °C until single colonies (for bacteria and yeast-like fungi) or growth (for hyphae-growing fungi) were visible. The single colonies were transferred to 5 mL TSB and incubated at 28 °C for 48 h to allow for recovery from freezing. These cultures were checked for contaminations by streaking subsamples onto TSA plates. To obtain a sufficient amount of bacterial biomass for the assays, bacteria were cultured at 28 °C for 48 h in 50-mL falcon tubes filled with 15 mL TSB or 500-mL Erlenmeyer flasks with 150 mL TSB. Bacterial cultures were harvested by centrifugation in 50-mL falcon tubes for 20 min at 4000 rpm. Growth was quantified by measurement of optical density at 600 nm.

### Nematode and substrate isolation and DNA extraction for MiSeq genotyping

Nematodes were isolated using three main methods: (1) the previously established standard approach, which uses the laboratory food *E. coli* as an attractant on Agar plates; (2) an approach based on a viscous medium in the absence of *E. coli*; and (3) a plate-based approach without *E. coli*. Substrate samples were collected from the German locations. In detail, several nematode samples from Germany were isolated following the previously established method that uses *E. coli* OP50 as attractant for worms [64]. Single *C. elegans* were individually transferred without any washing or sterilization step to fresh NGM plates, seeded with *E. coli*, and allowed to produce offspring via selfing, subsequently resulting in a growing worm population. These worm populations were maintained on *E. coli* for 2–3 weeks before they were frozen at −80 °C. Prior to DNA isolations, these samples were thawed and washed three times in M9 with 0.05 % Triton X-100 (M9-T). Total genomic DNA was extracted from these German samples using the PowerSoil DNA Isolation Kit (MO-BIO, Carlsbad, USA) following the manufacturer’s instructions with the addition of 0.4 mg/mL proteinase-K (Fermentas/Thermo Fisher Scientific, Waltham, USA) per spin column and subsequent incubation at 2 h at 55 °C before the bead-beating step. A Geno/Grinder 2000 (SPEX SamplePrep, Metuchen, USA) was used to homogenize the samples for 1 min at 1500 strokes/min. These samples were denoted “lab-enriched worms”.

For the German locations, we additionally isolated single nematodes directly from the substrates and without using *E. coli* as attractant. In these cases, environmental samples were spread out evenly on sterile 9-cm PFM agar plates and covered carefully with 20 mL sterile S-buffer containing 1.2 % hydroxymethylcellulose, 5 mg/mL cholesterol, 1 mM MgSO_4_, 1 mM CaCl_2_, and 0.1 % acetone. In this viscous medium, nematodes float to the surface within 1–2 h and can be collected under a dissecting microscope. For each sample, worms were aseptically collected in as little liquid as possible and transferred into 1 mL M9-T in a sterile 3-cm petri dish and washed three times by first incubation for more than 10 min in M9-T, followed by transfer into fresh M9 and repetition of the incubation step. To extract DNA, single worms were transferred immediately after washing to sterile wells of a 96-multi-well plate filled with 10 μL 2x Tris-EDTA buffer, pH 8 with 1 mg/μL proteinase K, and one to three 1-mm zirconium beads per well. Crude DNA was obtained by freezing the plates for at least 16 h at −80 °C, followed by bead-beating twice for 3 min at 1500 rpm in a Geno/Grinder and proteinase K digestion (1 h 55 °C, 20 min 98 °C). The nematode species was identified by diagnostic PCR [19] using 1 μL of crude DNA as template for a 15 μL duplex PCR reaction performed with GoTaq DNA Polymerase (Promega, Mannheim, Germany) and containing the primer pair nlp30 diagnostic for *C. elegans* (0.53 μM each) and Cre-ITS2 diagnostic for *C. remanei* (0.27 μM each). Cycling conditions included an initial denaturation step at 95 °C for 2 min, followed by 35 cycles of 95 °C for 45 s, 55 °C for 30 s, 72 °C for 1 min, and a final elongation step at 72 °C for 5 min. The species of the nematode was determined through the length of the PCR product, which was 154 bp for *C. elegans*/*nlp-30* and 300 bp for *C. remanei*/Cre-ITS2. Positively tested DNA was directly used for amplification of the bacterial V4 region. These samples were denoted “natural worms”.

Substrate samples, from which worms were successfully isolated from the German locations, were homogenized in liquid nitrogen, followed directly by DNA isolation, using the same methods as for the lab-enriched worms.

Nematodes from the French and Portuguese locations were processed in groups of 30–100 worms either directly after isolation from the substrates (denoted natural worms) or after they had been maintained for several weeks in the laboratory (lab-enriched worms). In both cases, worms were surface-sterilized following a previously described method [65]. From these worms, genomic DNA was isolated using a standard phenol-chloroform protocol with a 30-minute RNAse A step.

### Control of contamination with airborne microorganisms and removal of microbes from the worm surface

Contamination with airborne microorganisms in the laboratory environment can represent a serious problem for microbiome analyses. Therefore, we took precautions at several steps during processing of the natural samples. In particular, for the German material, all work with the natural samples was performed in a separate lab, in which no other research work was performed. This lab was repeatedly disinfected, resulting in high sterility conditions. The efficacy of these disinfection measures had been tested through positioning of open LB Agar plates, which usually did not contain any contaminations. It is worth noting that a large part of the Schulenburg group works with spore-forming bacteria (i.e., *Bacillus thuringiensis* [66, 67]), whereby the spores are easily transmitted through air and can easily cause contaminations. Contaminations with these spore-forming bacteria were never observed in this particular laboratory dedicated to work with natural *C. elegans* samples.

Furthermore, almost all steps of the DNA isolation protocol were done in a laminar flow cabinet (only excluding incubation in a thermoshaker and centrifugation). This laminar flow cabinet was restricted to work with the natural samples in the above mentioned lab, which itself was restricted to work with natural samples. Prior to DNA isolation, all required equipment was moved into the laminar flow cabinet. All equipment and the clean bench itself was very carefully disinfected using DNA Away (Molecular Bio-Products, Inc.), followed by UV irradiation. Only thereafter did we start with the DNA isolation protocol. In addition, DNA was always first isolated from worm samples and only thereafter for substrate samples, in order to avoid any possible carry-over contamination from putatively high-yield (substrate samples) to low-yield extractions (worm samples). After each DNA isolation session, we again carefully disinfected the laminar flow cabinet using DNA Away and UV irradiation, in order to keep the highest possible sterility level.

PCRs were always set-up under similarly high sterility conditions in the laminar flow cabinet. For each PCR performed, we always included a positive and, importantly, a negative control. The latter contained all reaction components excluding DNA. PCR results were always inspected through gel electrophoresis and, for selected cases, using Nanodrop measurements. The negative control never produced any amplification product. In addition to the above measures, our results argue against a contamination problem. If there was a general contamination problem, then these should have been most problematic for samples, for which one could expect few bacteria (and thus little bacterial DNA and low PCR yield); thus, all worm samples processed in our labs should have been affected to a similar extent. In contrast, we consistently identified significant differences in the identified microbiome from *C. elegans* versus *C. remanei*. Moreover, if there was a general contamination problem, then it is unlikely to consist of similar taxa in different labs in different countries. However, we consistently uncovered highly similar microbial communities for worm samples isolated in the Schulenburg lab in Germany and also worm samples isolated in the Félix lab in France. Moreover, these similarities were revealed from sample preparations, based on slightly different DNA isolation protocols, performed by different researchers in different countries. In consideration of these different observations, we deem it highly unlikely that the results were dominated by laboratory contaminations.

In addition to the measures against general microbial contaminations, we also minimized the presence of microorganisms from the surface of the nematodes in order to restrict our analysis to microbes from within the worm body. In particular, directly before DNA isolation for 16S genotyping of the German material, all nematode samples were washed three times in sterile M9-T. The same washing regime was also applied to the nematode samples characterized by differential interference contrast microscopy and FISH-staining combined with fluorescence microscopy (see below). During microscopy of these nematodes, we usually observed no microbes and, in very few cases, only single microbes attached to the cuticle. This is true for microscopy of natural nematode samples or the nematodes from the recolonization experiments. This was in strong contrast to the high abundance of microbes in the worm gut, again true for natural or experimental samples, as, for example, illustrated in Figs. 1d and 2c, and the movie in Additional file 6. Furthermore, the efficacy of the washing protocol was also assessed by comparing colony forming units of the pelleted worm samples (after the final washing step) with those of the supernatant of the final washing step, the wash buffer itself, and a control buffer, revealing significant removal of adherent bacteria, as presented in Additional file 7B. These observations, taken together, strongly suggest that rare cuticle colonizers are unlikely to have biased our main analyses of the worm’s microbiome.

### MiSeq sequencing of bacterial 16S ribosomal RNA

Bacterial 16S sequences were amplified using the Illumina variants of the primers 515 F and 806R as previously described [29]. Briefly, PCR reactions were carried out in duplicates of 25 μL volume, containing 10 ng template DNA and using the Phusion High Fidelity DNA Polymerase (Thermo Fisher Scientific, Waltham, USA) with the following conditions: initial denaturation at 98 °C for 3 min; 35 cycles of 98 °C for 10 s, 58 °C for 30 s, and 72 °C for 30 s; and final elongation at 72 °C for 10 min. Since the yield for single nematodes was sometimes low, the respective PCR amplicons were concentrated with NucleoFast 96 PCR ultrafiltration membranes (Macherey-Nagel, Düren, Germany) according to the manufacturer’s instructions. Amplicon libraries were prepared from pooled PCR reactions, standardized after QUBIT DNA quantification (French and Portuguese samples) or normalized with SequalPrep Normalization plates (Invitrogen, Carlsbad, USA; all German samples) according to the manufacturer’s instructions. The samples were sequenced on an Illumina MiSeq platform at either the CNRS facility Imagif (CNRS, Gif-sur-Yvette, France; all French and Portuguese samples) or the sequencing facility at the Kiel Institute for Clinical Molecular Biology (all German samples).

### MiSeq sequence quality control and processing

The obtained MiSeq paired-end reads were assembled and quality filtered using USEARCH 7.0.1090 [68]. Single reads with a minimum length of 200 were truncated if quality was lower than 5 and contigs of 200–270 bp length were formed by merging sequences with at least 200 bp overlap and without allowed mismatches Resulting contigs with an overall expected error probability of ≥ 0.1 were removed. We used mothur 1.33.3 [69] for downstream processing of reads. In brief, reads were aligned to a reference based on the Silva V119 alignment [70] using a k-mer based Needleman algorithm. Sequence chimeras were removed with UCHIME [71]. Sequences were assigned a taxonomy using a Bayesian classifier [72] on the RDP trainset with an 80 % bootstrap confidence. In all subsequent analyses, a normalized subset of 2000 sequences was used. OTUs were clustered at 97 % similarity and classified based on the previously created taxonomy. Metrics for alpha and beta diversity were calculated based on the clustered OTUs with the mothur-implemented methods and visualized with R.

### Statistical analysis of MiSeq 16S ribosomal RNA genotyping

We conducted statistical analysis using R. We compared strain frequencies using non-parametric Wilcoxon rank sum tests. Microbial alpha and beta diversity metrics were calculated with the methods implemented in mothur 1.33.3 [69]. We analyzed the alpha diversity of the wild *Caenorhabditis* samples with generalized linear models assuming a Gaussian error distribution and considering host species and sample type, location, and environment as possible explanatory variables. Beta diversity dissimilarities were analyzed using the *vegan*-implementations of the ADONIS function for non-parametric permutational multivariate analysis of variance [30] and for unconstrained ordination by principle coordinates (principal coordinate analysis). Model selection for canonical correspondence analysis [73] of OTU abundance aimed at explaining most of the inertia while retaining the least amount of explanatory variables. We considered sample type, location, and environment as possible explanatory variables.

### Establishment and analysis of an experimental microbiome

The experimental microbiome consisted of 14 different bacterial isolates (Additional file 1: Tables S1-3 and S1-9). Of these, nine were representatives of the 15 most abundant genera, which could be identified in the MiSeq genotyping analysis for native *C. elegans* and for which culturable isolates were available (Additional file 1: Table S1-3; note that several genera were found to be abundant more than once). We added representatives of two additional abundant genera, namely *Comamonas* and *Rhodococcus*, both of which belong to the top 25 most abundant genera, that could be identified through MiSeq genotyping and for which isolates were available (Additional file 1: Table S1-3). We further included representatives of three additional genera that belonged to abundant and taxonomically distinct bacterial orders such as *Achromobacter* (order Burkholderiales), *Bacillus* (order Bacillales), and *Microbacterium* (order Actinomycetales; Additional file 1: Table S1-3).

Synchronized *C. elegans* L1 larvae were raised at 20 °C on 9-cm agar plates seeded with 400 μL of a suspension containing the 14 bacterial isolates, with a final OD_600_ per bacterium of 5. Nematodes and bacterial lawns were harvested after 48 h (L4 larvae) and 72 h (adults) with M9-T + 30 mM NaN_3_ to anesthetize worms for a short period and prevent the intake of bleach during the following washing step. To remove bacteria outside of the nematode gut, worms were washed by placing them onto a 10-μm filter, followed by addition of M9-T mixed with 1:100 bleach solution [16], 5 min incubation, and subsequent centrifugation. Thereafter, worms were washed twice more in M9 only. This procedure allowed for an efficient removal of bacteria attached to the worm’s surface (Additional file 7B), while retaining nematodes alive. For performance of the experiment, we used number codes for the treatments to avoid observer bias, treatments were spatially randomized in the incubator and assessed in a randomized order to minimize the influence of random environmental effects, and all treatments were replicated eight times. Worm and lawn samples were then processed and statistically analyzed following the details given above for the wild *C. elegans* samples.

Inference of bacterial frequencies was based on a customized alignment consisting of 16S sequences from all used bacteria, which were obtained by Sanger-sequencing. For safety, we additionally performed a BLAST search for all MiSeq sequences obtained in order to assess whether the obtained MiSeq sequences were indeed the best match for the used strains. This additional analysis revealed a mismatch for one of the major OTUs, where a sequence was most similar to the *Ochrobactrum* sequence in our reference alignment whereas it was identified by BLAST to belong to the genus *Sphingomonas* (related NCBI Accessions gb|HM438390.1| or gb|AF408323.1|). In order to avoid any biases in the results, we decided to exclude any sample from further analysis which had more than 1.5 % of all reads showing highest BLAST similarity to the above *Sphingomonas* sp. This was the case for two out of eight replicates of our L4 staged nematode samples of strain MY379 (i.e., the treatment group “MY379 L4 worms”), and also single replicates from the groups “N2 L4 worms”, “N2 adult worms”, “MY379 adult worms”, and “MY316 L4 worms”. Furthermore, three samples of “MY379 L4 worms” and single samples of “N2 L4 lawn” and “MY316 L4 worms” did not have sufficient sequence coverage (less than 1000 sequence reads after quality control) and were therefore excluded. After exclusion of these cases, only three replicates remained for treatment “MY379 L4 worms”, which would have made comparison with the other treatments unbalanced. Therefore, we excluded this particular treatment from all further statistical analysis and only showed the results for the remaining three replicates in Fig. 2a. An overview of replicate numbers per treatment and the included samples per treatment are provided in Additional file 1: Tables S1-10 and S1-11.

### Population size analysis

Population size was used as a proxy for evolutionary fitness and the assay generally followed the previously established protocol [74]. We assessed the effects of the same 14 bacterial isolates included in the experimental microbiome. We considered 10 additional isolates from the same genera, in order to obtain a first indication of within-genus bacterial variation (see Tables S1-3 and S1-9 for an overview of the considered isolates). Three synchronized L4 hermaphrodites were placed with a platinum wire on a 6-cm NGM-agar plate seeded with 400 μL of the respective food bacterium with an OD_600_ of 10 or the experimental microbiota mix (see above). After 5 days at 20 °C, the worm populations were washed with 2 mL M9-T and frozen at −20 °C. The number of worms in 5 μL of suspension was counted three times to calculate the total number of worms per plate. To correct for the uneven liquid loss during washing, we weighted the test tubes afterwards and corrected our calculations accordingly.

The population growth assays always consisted of 10–11 independent replicates per treatment. Treatments were randomized and coded by numbers to avoid any observer bias. Normality of the data was examined using quantile plots and the Shapiro–Wilk normality test. All treatments were then compared to the *E. coli* OP50 control using Wilcoxon’s rank sum test and *P* values were corrected for multiple testing using the false discovery rate [75].

### Fluorescence *in situ* hybridization assay for bacterial quantification

Synchronized *C. elegans* L1 larvae were raised on 9-cm agar plates seeded with 400 μL of the respective test bacterium with an OD_600_ of 10 for 72 h at 20 °C until adulthood (time point 0 h). Populations were then split, washed three times in M9-T, and transferred to a PFM-plate containing either the same food or no food (TSB mock inoculated). 0 h and 24 h after transfer, nematodes were harvested, washed, and fixed in 3 % formaldehyde for 1 h at room temperature (RT). FISH was essentially performed as previously described [76], based on the probe EUB338, which binds to ribosomal RNA and should thus particularly highlight alive cells, in which RNA had not yet been degraded by exogenous RNAses. The exact protocol was modified to increase hybridization efficacy in nematodes. In particular, worms were washed for 30 min in PBS and 30 min in 50 % EtOH/PBS at RT. Samples were collected in 250 μL hybridization buffer (20 mM Tris, 0.1 % SDS, 900 mM NaCl) and heated at 80 °C for 5 min before adding the probe. Then, 2.5 μL 100 μM probe EUB338 [76], 5’-labeled with Cy3, was added to the buffer and staining commenced for 20 min at 55 °C. Samples were washed for 30 min in hybridization buffer containing no probe at 55 °C and 30 min in PBS at RT. Stained samples were transferred to 90 % glycerol/PBS and analyzed with a confocal LSM700 microscope using a 555 nM solid-state laser. We quantified bacterial load as ranked prevalence of stained bacteria in the pharynx and the anterior and posterior intestine, separated by the position of the vulva, using four categories: (0) absent, (1) single cells, countable, (2) clumps of cells, too many to count, and (3) region is completely filled. For each replicate, we assessed 10 worms and then calculated the mean categorical rank for these worms and for each three-body region. The evaluation of worms was performed for coded samples in fully randomized order of treatments by an experienced technician, otherwise not connected to the project. This assay was performed in three independent biological replicates.

### Anti-fungal activity

The fungal isolates *Mucor* sp. MYf197, *Dipodascus* sp. MYf82, *Mortierella* sp. MYf35, *Penicillium* sp. MYf125, *Fusarium* so. MYf198, and *Trichoderma* sp. MYf192 were allowed to completely overgrow a TSA plate at 25 °C. A 0.5 mm^2^ piece from the center of the stock plates was aseptically transferred to the center of a TSA plate seeded with 400 μL of either the *Pseudomonas* isolates MYb11, MYb187, or MYb193, or *E. coli* OP50 with an OD_600_ of 10. We scored the diameter of the growing fungus over 3 days and calculated the relative growth in comparison to the mean fungal growth on *E. coli* OP50.

The anti-fungal effect of *Pseudomonas* co-isolates was performed in triplicate and analyzed within the linear model framework with the relative growth as response variable and the fungal and bacterial strains as explanatory variables. All treatments were randomized and coded by numbers to avoid any observer bias.

### Survival on *Drechmeria coniospora*

Synchronized *C. elegans* L1 larvae were grown at 20 °C on 9-cm agar plates seeded with 400 μL of a suspension containing either *Pseudomonas* MYb11 or *E. coli* OP50 with an OD_600_ of 5 until they reached the L4 stage. Subsequently, 30 L4 larvae were transferred to fresh plates seeded with a mixture of either bacterium and additionally conidia of the fungal pathogen *Drechmeria coniospora* (prepared as previously described [77, 78]) or M9-T buffer (controls). Nematode survival was scored after 48 h at 25 °C. The assay was performed in triplicate with randomized order of treatments and number-coded treatments to avoid observer bias. The significance of worm mortality on *D. coniospora* relative to M9 controls was assessed using Wilcoxon rank sum tests.

### Statistical analysis packages

We utilized R [79] for all statistical analyses, using the following non-base packages: *ggplot2* [80], *gridBase* [81], *gridExtra* [82], *gtable* [83], *lawstat* [84], *rcolorbrewer* [85], *reshape* [86], *rgl* [87], *scales* [88], and *vegan* [31].

## Availability of data and materials

The original Miseq data of the native microbiome and the experimental microbiome is available from the European Nucleotide Archive under the accessions ERP014530 and ERP014569, respectively. Bacterial 16S ribosomal RNA gene sequences and fungal ribosomal internal transcribed spacer sequences for the 24 bacterial and six fungal isolates, respectively, which were characterized in more detail, are available from GenBank under the accessions KU902420-KU902449. The ribosomal sequences for all other isolates are available from GenBank under accession numbers KX079706–KX079868. Phenotypic data for population size analysis and antifungal analysis is provided in Additional file 1: Tables S1-17, S1-18, S1-20, S1-22, and S1-24. The bacterial, fungal, and nematode isolates studied herein are available from the Schulenburg lab on request.

## Additional files

Additional file 1: Data and results from statistical analysis of the native and experimental microbiome of *C. elegans*. This document contains a total of 23 tables as sheets of an Excel file, which summarize the material used and the results of the statistical analyses. **Table S1-1.** List of samples used for analysis of the *Caenorhabditis* native microbiome. **Table S1-2.** Summary of sample numbers used for analysis of the *Caenorhabditis* native microbiome. **Table S1-3.** Top 100 operational taxonomic units (OTUs) of the native microbiome of *Caenorhabditis elegans*. **Table S1-4.** Top 100 OTUs of the native microbiome of *Caenorhabditis remanei*. **Table S1-5.** Top 100 OTUs of the native microbiome of *Caenorhabditis briggsae*. **Table S1-6.** Statistical comparison of bacterial taxa abundances among sample types for the native *Caenorhabditis* microbiome. **Table S1-7.** Statistical evaluation of the factors for the canonical correspondence analysis of the native *Caenorhabditis* microbiome. **Table S1-8.** Statistical evaluation of the factors for the multivariate analysis of variance of the native *Caenorhabditis* microbiome. **Table S1-9.** List of bacterial and fungal isolates from natural *C. elegans* samples or corresponding substrates. **Table S1-10.** Overview of sample sizes for analysis of the experimental microbiome. **Table S1-11.** Overview of included and excluded samples in the analysis of the experimental microbiome. **Table S1-12.** Statistical comparison of bacterial taxa abundances among sample types for the experimental *C. elegans* microbiome. **Table S1-13.** Statistical evaluation of the factors for the canonical correspondence analysis of the experimental *C. elegans* microbiome. **Table S1-14.** Statistical evaluation of the factors for the multivariate analysis of variance of the experimental *C. elegans* microbiome. **Table S1-15.** Statistical comparison of bacterial taxa abundances among sample types for the second experiment with the experimental *C. elegans* microbiome. **Table S1-16.** Statistical evaluation of the factors for the canonical correspondence analysis of the second experiment with the experimental *C. elegans* microbiome. **Table S1-17.** Raw data for the bacterial colonization experiment. **Table S1-18.** Raw data for the population growth experiment with the experimental microbiome under different conditions. **Table S1-19.** Statistical analysis of *C. elegans* population growth on the experimental microbiome under different conditions. **Table S1-20.** Raw data for the population growth experiment with individual bacterial isolates. **Table S1-21.** Statistical analysis of *C. elegans* population growth on individual bacterial isolates. **Table S1-22.** Raw data for the analysis of bacterial effects on fungal growth. **Table S1-23.** General linear model analysis of antifungal effects of three *Pseudomonas* isolates. **Table S1-24.** Raw data for the bacterial effect on Drechmeria-induced worm killing. **Table S1-25.** Statistical analysis of an in vivo antifungal effect of *Pseudomonas M*Yb11. (XLSX 116 kb) Additional file 2: A three-dimensional movie visualization of the canonical correspondence analysis of bacterial operational taxonomic units-abundance in natural *Caenorhabditis* isolates and their substrates. Sample types and nematode species are indicated by colors. (MP4 558 kb) Additional file 3: Movie visualization of the principal coordinate analyses (PCoA) of the native *C. elegans* microbiome. PCoA based on Jaccard dissimilarity (top left), Bray–Curtis dissimilarity (top right), Unweighted UniFrac distances (bottom left), and Weighted UniFrac distances (bottom right). The panels for one of the dissimilarity measures show the first three axes of PCoA. The analysis considered nematode species (*C. elegans*, *C. remanei*, or *C. briggsae*), sample type (natural worms, lab-enriched worms, substrate), and substrate types (stem, fruit, compost, and vector) as factors. The statistics are given in Additional file 1: Tables S1-6, S1-7, and S1-8. (MP4 3434 kb) Additional file 4: Frequencies of microbial isolates from natural *Caenorhabditis* samples or their substrates. (A) Bacterial and (B) fungal isolates (Additional file 1: Table S1-9). The material was used to select specific isolates as representatives of the abundant genera from the native *C. elegans* microbiome (see Fig. 1 of the main text and Additional file 1: Table S1-3). We characterized bacteria through partial 16S ribosomal RNA gene sequences and fungi through ribosomal internal transcribed spacer sequences. The approximate taxonomic position of the isolates was subsequently assessed with the help of a BLAST-based similarity analysis, which is sufficient for an approximate classification of the isolates, especially at higher taxonomic levels and as required at this particular step, even though exact species designations may not always be correct. (PNG 845 kb) Additional file 5: Phylogenetic analyses of the 24 bacterial and six fungal isolates used in this study. Maximum likelihood-based phylogenetic trees were based on either GTR models (Actinobacteria, Firmicutes, Bacteroidetes, and the fungi) or HKY models (joint analysis of Alpha- and Beta-Proteobacteria, as well as Gamma-Proteobacteria). The new isolates are given in blue font, whereas those from public databases in black. For the published bacterial strains, the first number is the accession number of the Ribosomal Database Project (RDP, [58]) and the last is its GenBank accession. For the published fungal strains, the last number refers to the respective GenBank accession. The resulting phylogenies were generally in agreement with the BLAST-based assignments – with two exceptions: the BLAST assigned genus *Serpens* has been merged into *Pseudomonas* [89], and the isolates of *Galactomyces* have been reclassified as *Dipodascus* [90], thus we renamed our isolates accordingly. The six trees show branch-lengths according to the inferred evolutionary distance. Bootstrap support from 1000 replicates are indicated if above 75 % (gray circles), above 95 % (red circles), or above 99 % (orange circles). (PDF 32 kb) Additional file 6: A three-dimensional movie visualization of *C. elegans* inhabited by its experimental microbiome. Confocal laser scanning micrograph of a nematode containing bacteria visualized through fluorescence *in situ* hybridization with the general eubacterial probe EUB338 in red. (MP4 3241 kb) Additional file 7: Bacterial abundance in *C. elegans* nematodes recolonized with an experimental microbiome. (A) Average bacterial read abundance shown separately for each taxon in box plots. We inoculated sterile nematode eggs with a mixture of 14 bacteria to study the recolonization of three *C. elegans* strains (N2, MY316, MY379) during development (L4, Adult). We used MiSeq sequencing to quantify the relative bacterial abundance for nematodes and corresponding lawns. (B) Bacterial load of worms assessed during DNA isolation for MiSeq sequencing. To separate nematodes from bacteria, we centrifuged worms on filters followed by mild bleaching (see Methods). When applied to a mixed lawn without worms, this treatment (wash buffer) reduced the number of residual bacteria to negative control levels (control buffer). This treatment also removed adhering bacteria from the outside of worms. Worms treated with this subsequent wash step (worm buffer), when processed, showed lower colony forming units by orders of magnitude compared to the washed worms alone (worms pellet). (PNG 1328 kb) Additional file 8: A three-dimensional movie visualization of the canonical correspondence analysis of the experimental microbiome of *C. elegans*. Sample types, nematode developmental stage, and genotype are indicated by colors and symbols. (MP4 435 kb) Additional file 9: Movie visualization of the principal coordinate analyses (PCoA) of *C. elegans* nematodes recolonized with an experimental microbiome. PCoA based on Jaccard dissimilarity (top left), Bray–Curtis dissimilarity (top right), Unweighted UniFrac distances (bottom left), and Weighted UniFrac distances (bottom right). The analysis considered sample type (nematode or lawn), developmental stage (L4 or adult), and *C. elegans* genotype (N2, MY316, MY379) as factors. The statistics are given in Additional file 1: Tables S1-12, S1-13, and S1-14. (MP4 2306 kb)
