# Supplementary figures and images for: The native microbiome of the nematode Caenorhabditis elegans: gateway to a new host-microbiome model
Source: BMC Biol. 2016 May 9;14:38. doi: 10.1186/s12915-016-0258-1 (PMC4860760; doi:10.1186/s12915-016-0258-1)

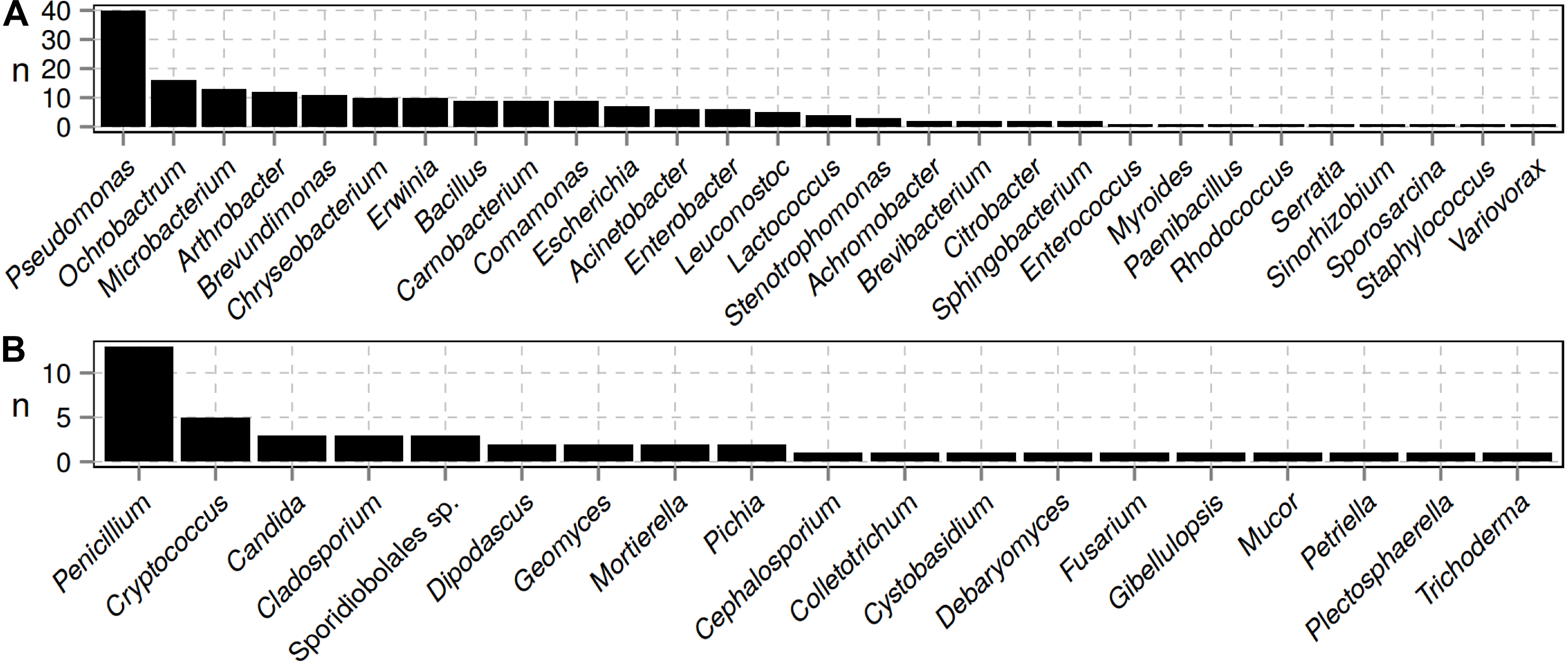

Supplement: Additional file 4: — Frequencies of microbial isolates from natural Caenorhabditis samples or their substrates. (A) Bacterial and (B) fungal isolates (Additional file 1: Table S1-9). The material was used to select specific isolates as representatives of the abundant genera from the native C. elegans microbiome (see Fig. 1 of the main text and Additional file 1: Table S1-3). We characterized bacteria through partial 16S ribosomal RNA gene sequences and fungi through ribosomal internal transcribed spacer sequences. The approximate taxonomic position of the isolates was subsequently assessed with the help of a BLAST-based similarity analysis, which is sufficient for an approximate classification of the isolates, especially at higher taxonomic levels and as required at this particular step, even though exact species designations may not always be correct. (PNG 845 kb) [file 12915_2016_258_MOESM4_ESM.png]

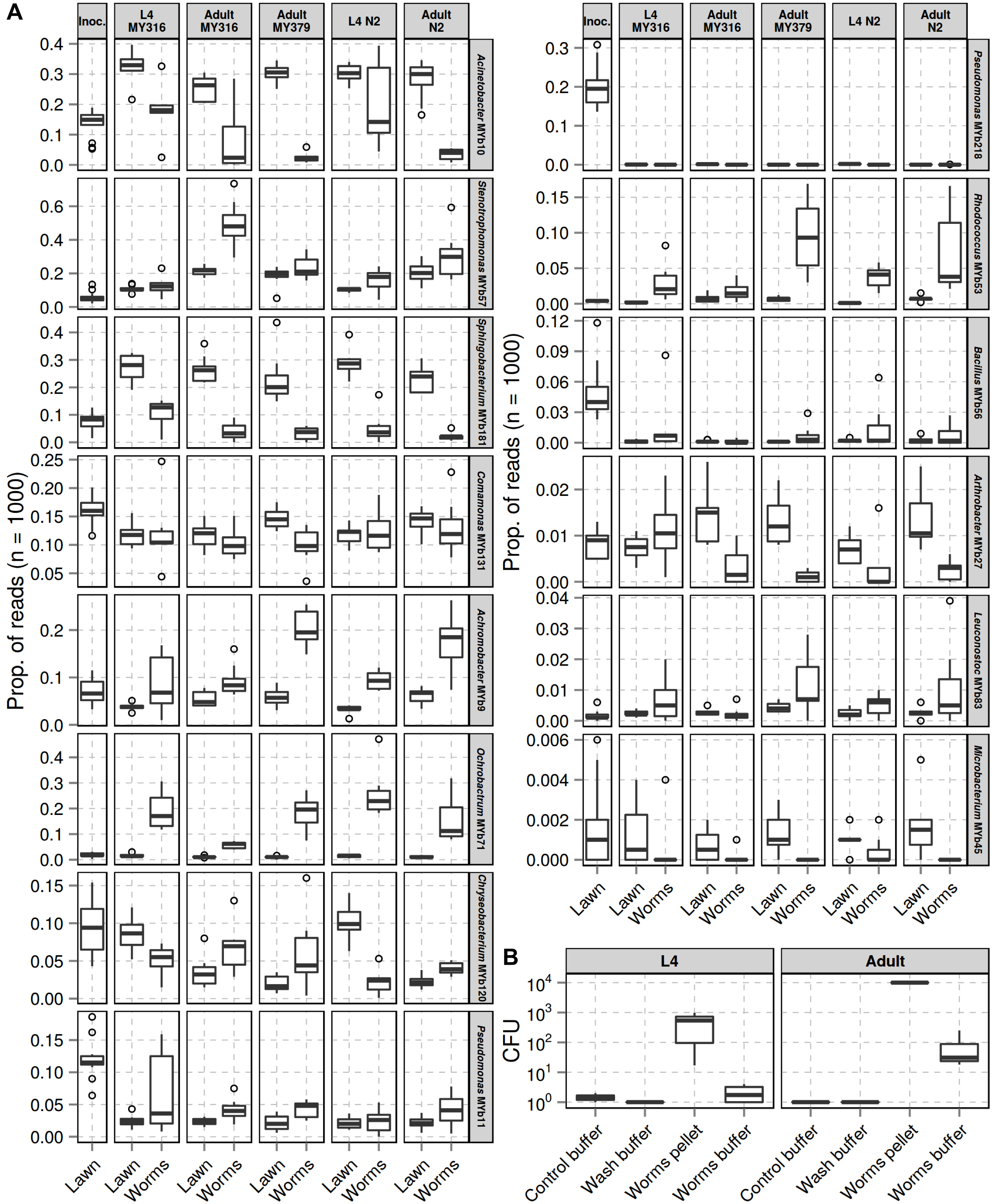

Supplement: Additional file 7: — Bacterial abundance in C. elegans nematodes recolonized with an experimental microbiome. (A) Average bacterial read abundance shown separately for each taxon in box plots. We inoculated sterile nematode eggs with a mixture of 14 bacteria to study the recolonization of three C. elegans strains (N2, MY316, MY379) during development (L4, Adult). We used MiSeq sequencing to quantify the relative bacterial abundance for nematodes and corresponding lawns. (B) Bacterial load of worms assessed during DNA isolation for MiSeq sequencing. To separate nematodes from bacteria, we centrifuged worms on filters followed by mild bleaching (see Methods). When applied to a mixed lawn without worms, this treatment (wash buffer) reduced the number of residual bacteria to negative control levels (control buffer). This treatment also removed adhering bacteria from the outside of worms. Worms treated with this subsequent wash step (worm buffer), when processed, showed lower colony forming units by orders of magnitude compared to the washed worms alone (worms pellet). (PNG 1328 kb) [file 12915_2016_258_MOESM7_ESM.png]
