# Supplementary material for: The native microbiome of the nematode Caenorhabditis elegans: gateway to a new host-microbiome model
Source: BMC Biol. 2016 May 9;14:38. doi: 10.1186/s12915-016-0258-1 (PMC4860760; doi:10.1186/s12915-016-0258-1)
Supplement: Additional file 5: — Phylogenetic analyses of the 24 bacterial and six fungal isolates used in this study. Maximum likelihood-based phylogenetic trees were based on either GTR models (Actinobacteria, Firmicutes, Bacteroidetes, and the fungi) or HKY models (joint analysis of Alpha- and Beta-Proteobacteria, as well as Gamma-Proteobacteria). The new isolates are given in blue font, whereas those from public databases in black. For the published bacterial strains, the first number is the accession number of the Ribosomal Database Project (RDP, [58]) and the last is its GenBank accession. For the published fungal strains, the last number refers to the respective GenBank accession. The resulting phylogenies were generally in agreement with the BLAST-based assignments – with two exceptions: the BLAST assigned genus Serpens has been merged into Pseudomonas [89], and the isolates of Galactomyces have been reclassified as Dipodascus [90], thus we renamed our isolates accordingly. The six trees show branch-lengths according to the inferred evolutionary distance. Bootstrap support from 1000 replicates are indicated if above 75 % (gray circles), above 95 % (red circles), or above 99 % (orange circles). (PDF 32 kb) [file 12915_2016_258_MOESM5_ESM.pdf]

Actinobacteria

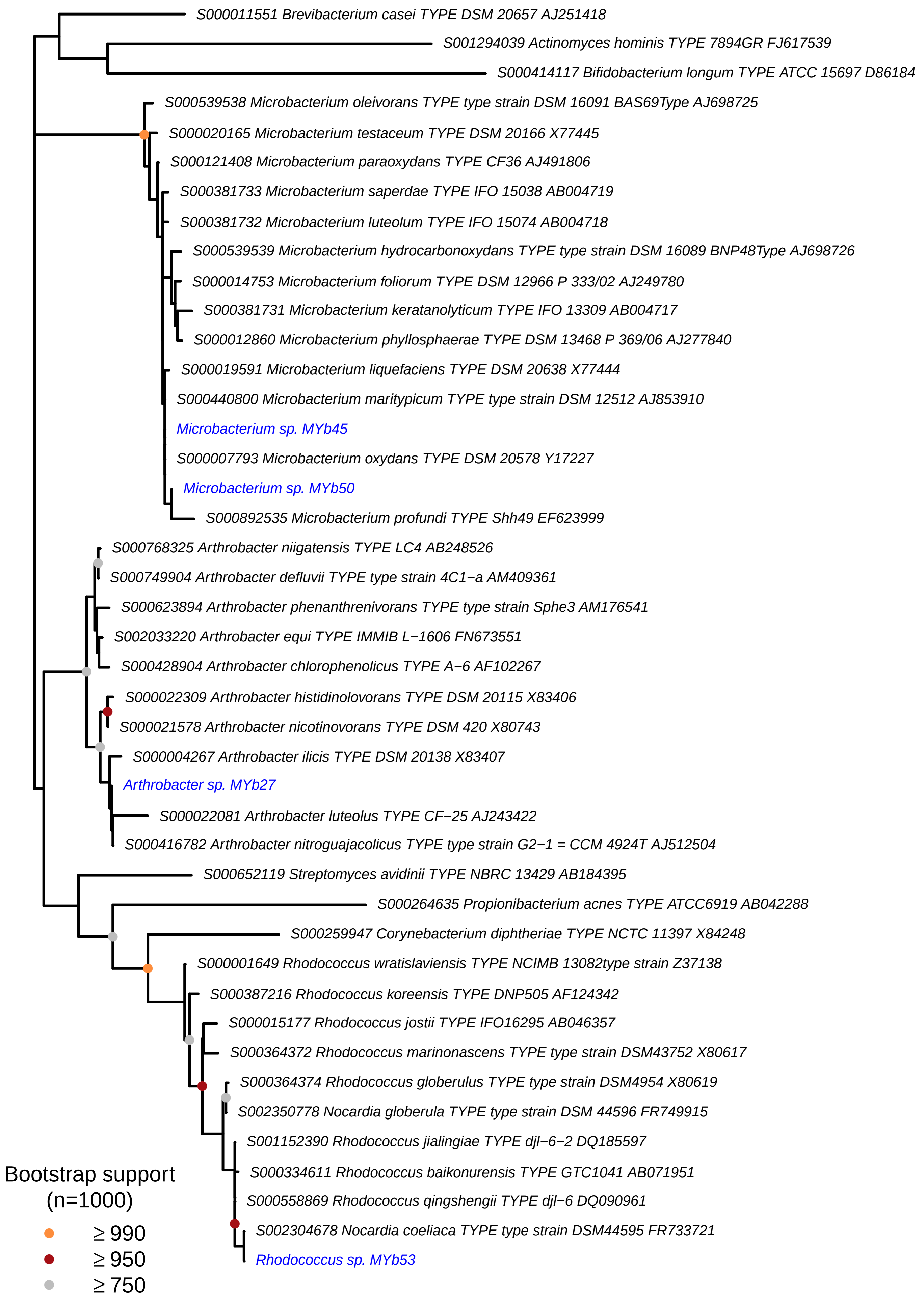

Firmicutes

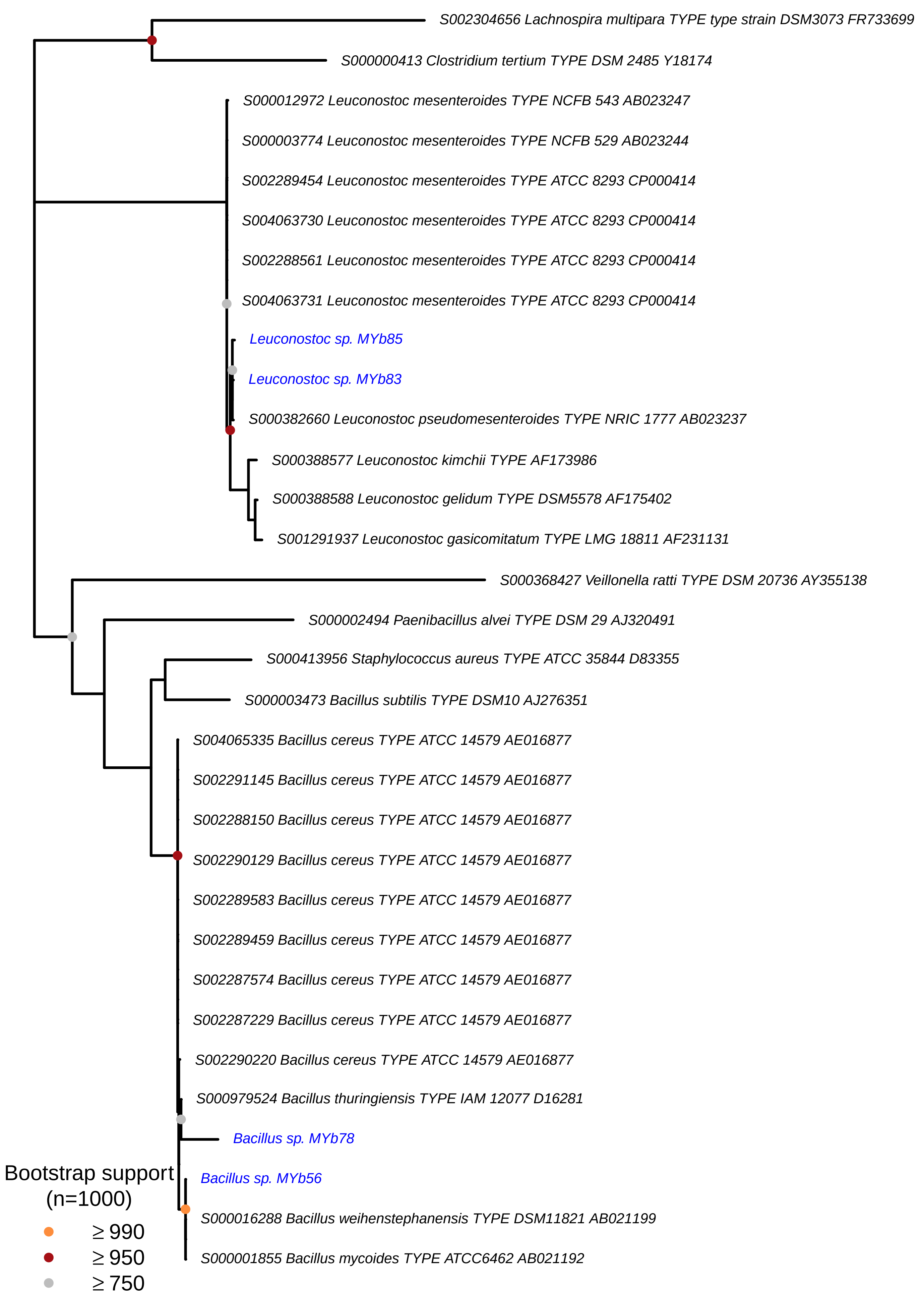
S002304656 Lachnospira multipara TYPE type strain DSM3073 FR733699
S000000413 Clostridium tertium TYPE DSM 2485 Y18174
S000012972 Leuconostoc mesenteroides TYPE NCFB 543 AB023247
S000003774 Leuconostoc mesenteroides TYPE NCFB 529 AB023244
S002289454 Leuconostoc mesenteroides TYPE ATCC 8293 CP000414
S004063730 Leuconostoc mesenteroides TYPE ATCC 8293 CP000414
S002288561 Leuconostoc mesenteroides TYPE ATCC 8293 CP000414
S004063731 Leuconostoc mesenteroides TYPE ATCC 8293 CP000414
Leuconostoc sp. MYb85
Leuconostoc sp. MYb83
S000382660 Leuconostoc pseudomesenteroides TYPE NRIC 1777 AB023237
S000388577 Leuconostoc kimchii TYPE AF173986
S000388588 Leuconostoc gelidum TYPE DSM5578 AF175402
S001291937 Leuconostoc gasicomitatum TYPE LMG 18811 AF231131
S000368427 Veillonella ratti TYPE DSM 20736 AY355138
S000002494 Paenibacillus alvei TYPE DSM 29 AJ320491
S000413956 Staphylococcus aureus TYPE ATCC 35844 D83355
S000003473 Bacillus subtilis TYPE DSM10 AJ276351
S004065335 Bacillus cereus TYPE ATCC 14579 AE016877
S002291145 Bacillus cereus TYPE ATCC 14579 AE016877
S002288150 Bacillus cereus TYPE ATCC 14579 AE016877
S002290129 Bacillus cereus TYPE ATCC 14579 AE016877
S002289583 Bacillus cereus TYPE ATCC 14579 AE016877
S002289459 Bacillus cereus TYPE ATCC 14579 AE016877
S002287574 Bacillus cereus TYPE ATCC 14579 AE016877
S002287229 Bacillus cereus TYPE ATCC 14579 AE016877
S002290220 Bacillus cereus TYPE ATCC 14579 AE016877
S000979524 Bacillus thuringiensis TYPE IAM 12077 D16281
Bacillus sp. MYb78
Bacillus sp. MYb56
S000016288 Bacillus weihenstephanensis TYPE DSM11821 AB021199
S000001855 Bacillus mycoides TYPE ATCC6462 AB021192
Bootstrap support
(n=1000)
≥ 990
≥ 950
≥ 750

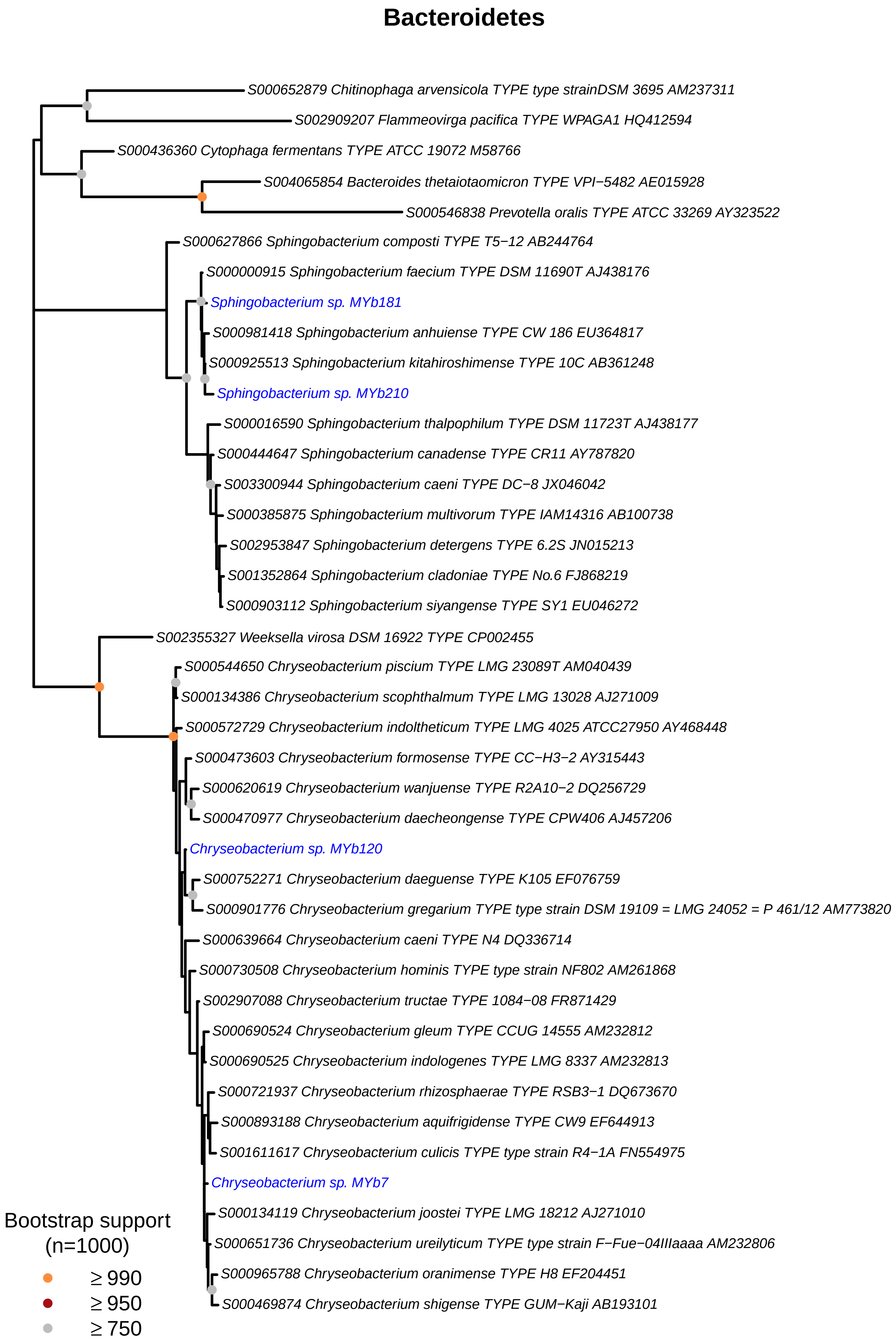
Bacteroidetes
S000652879 Chitinophaga arvensicola TYPE type strainDSM 3695 AM237311
S002909207 Flammeovirga pacifica TYPE WPAGA1 HQ412594
S000436360 Cytophaga fermentans TYPE ATCC 19072 M58766
S004065854 Bacteroides thetaiotaomicron TYPE VPI−5482 AE015928
S000546838 Prevotella oralis TYPE ATCC 33269 AY323522
S000627866 Sphingobacterium composti TYPE T5−12 AB244764
S000000915 Sphingobacterium faecium TYPE DSM 11690T AJ438176
Sphingobacterium sp. MYb181
S000981418 Sphingobacterium anhuiense TYPE CW 186 EU364817
S000925513 Sphingobacterium kitahiroshimense TYPE 10C AB361248
Sphingobacterium sp. MYb210
S000016590 Sphingobacterium thalpophilum TYPE DSM 11723T AJ438177
S000444647 Sphingobacterium canadense TYPE CR11 AY787820
S003300944 Sphingobacterium caeni TYPE DC−8 JX046042
S000385875 Sphingobacterium multivorum TYPE IAM14316 AB100738
S002953847 Sphingobacterium detergens TYPE 6.2S JN015213
S001352864 Sphingobacterium cladoniae TYPE No.6 FJ868219
S000903112 Sphingobacterium siyangense TYPE SY1 EU046272
S002355327 Weeksella virosa DSM 16922 TYPE CP002455
S000544650 Chryseobacterium piscium TYPE LMG 23089T AM040439
S000134386 Chryseobacterium scophthalmum TYPE LMG 13028 AJ271009
S000572729 Chryseobacterium indoltheticum TYPE LMG 4025 ATCC27950 AY468448
S000473603 Chryseobacterium formosense TYPE CC−H3−2 AY315443
S000620619 Chryseobacterium wanjuense TYPE R2A10−2 DQ256729
S000470977 Chryseobacterium daecheongense TYPE CPW406 AJ457206
Chryseobacterium sp. MYb120
S000752271 Chryseobacterium daeguense TYPE K105 EF076759
S000901776 Chryseobacterium gregarium TYPE type strain DSM 19109 = LMG 24052 = P 461/12 AM773820
S000639664 Chryseobacterium caeni TYPE N4 DQ336714
S000730508 Chryseobacterium hominis TYPE type strain NF802 AM261868
S002907088 Chryseobacterium tructae TYPE 1084−08 FR871429
S000690524 Chryseobacterium gleum TYPE CCUG 14555 AM232812
S000690525 Chryseobacterium indologenes TYPE LMG 8337 AM232813
S000721937 Chryseobacterium rhizosphaerae TYPE RSB3−1 DQ673670
S000893188 Chryseobacterium aquifrigidense TYPE CW9 EF644913
S001611617 Chryseobacterium culicis TYPE type strain R4−1A FN554975
Chryseobacterium sp. MYb7
S000134119 Chryseobacterium joostei TYPE LMG 18212 AJ271010
S000651736 Chryseobacterium ureilyticum TYPE type strain F−Fue−04IIIaaaa AM232806
S000965788 Chryseobacterium oranimense TYPE H8 EF204451
S000469874 Chryseobacterium shigense TYPE GUM−Kaji AB193101
Bootstrap support
(n=1000)
≥ 990
≥ 950
≥ 750

Alphaproteobacteria & Betaproteobacteria

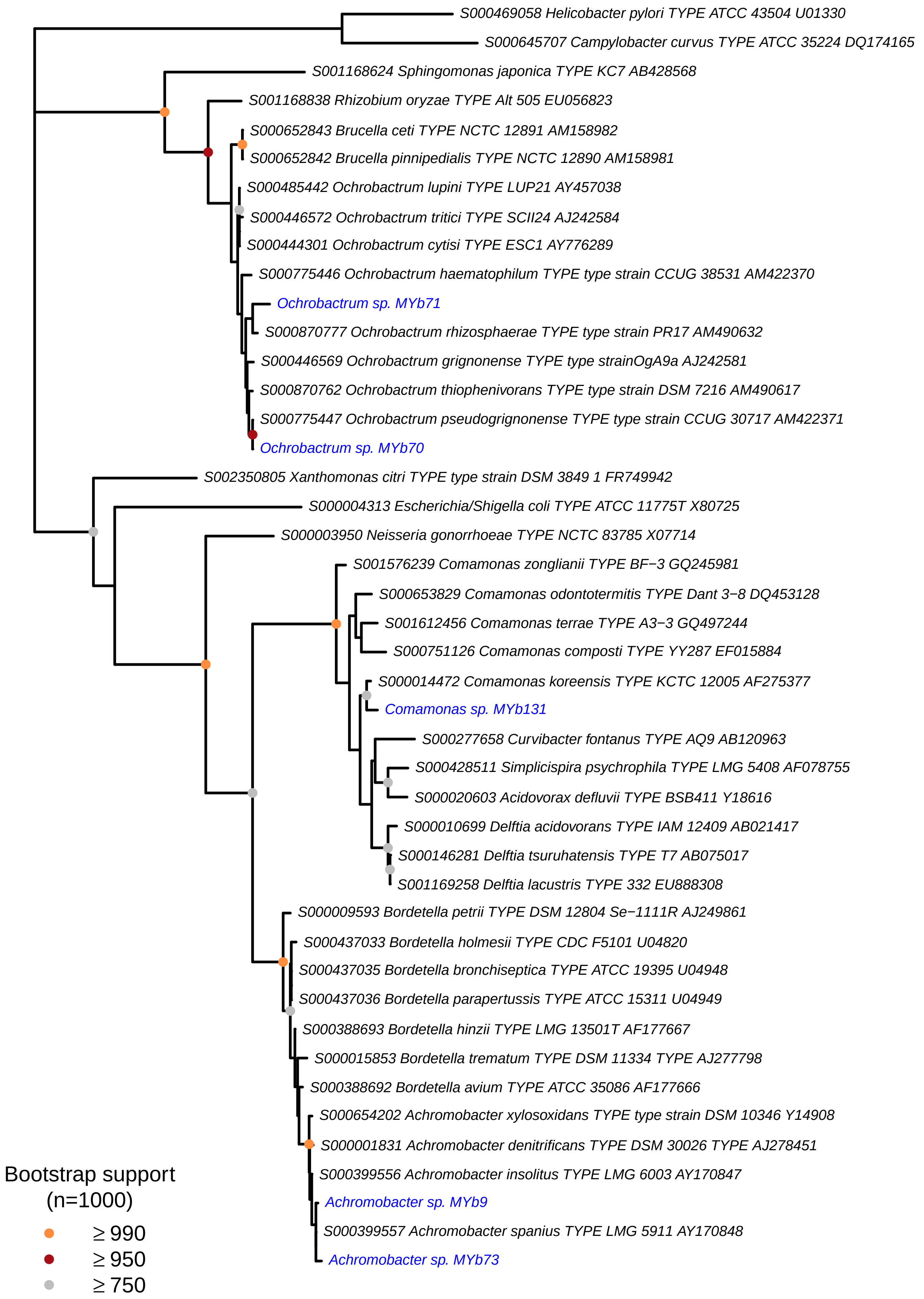

Gammaproteobacteria

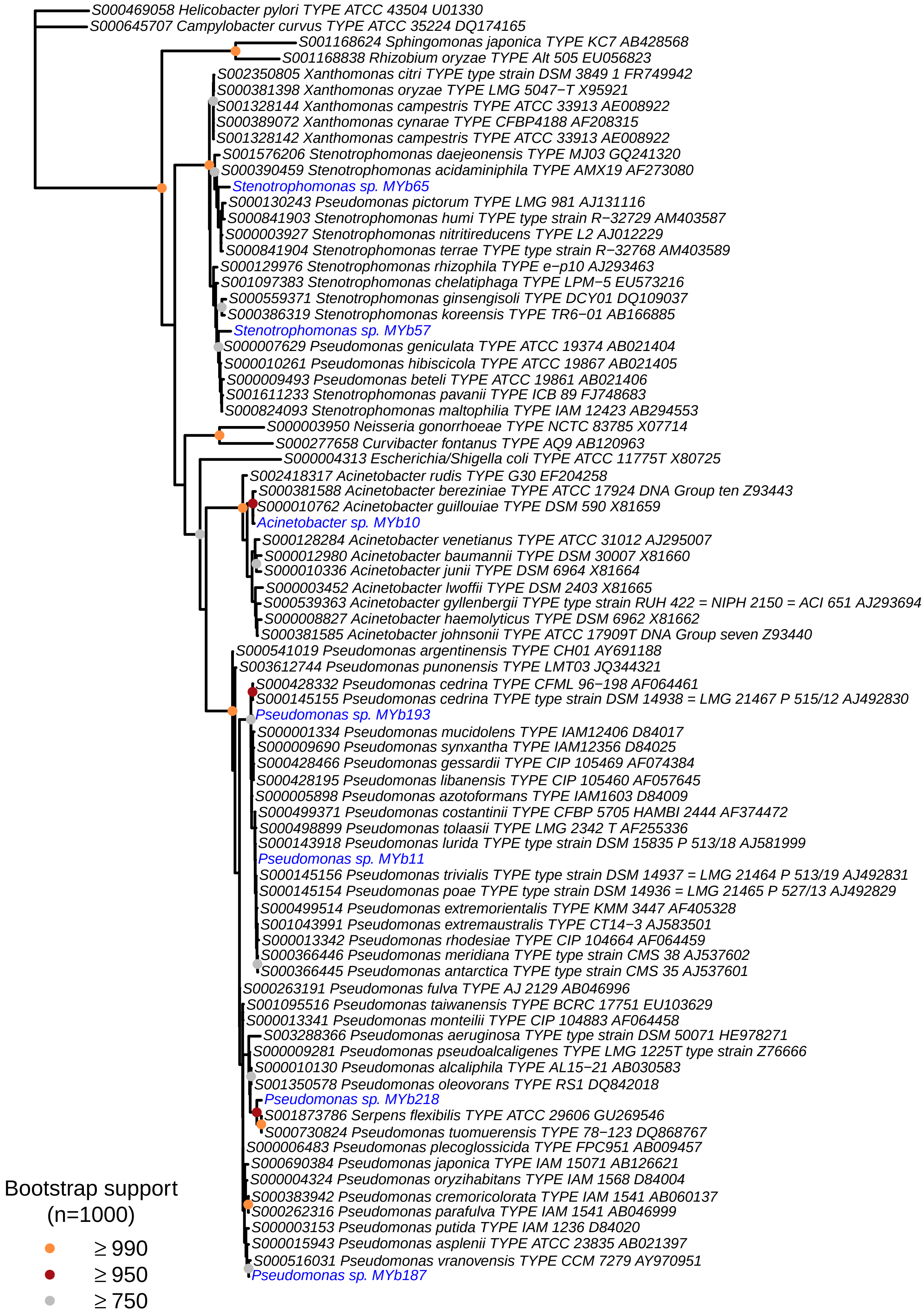

Fungi

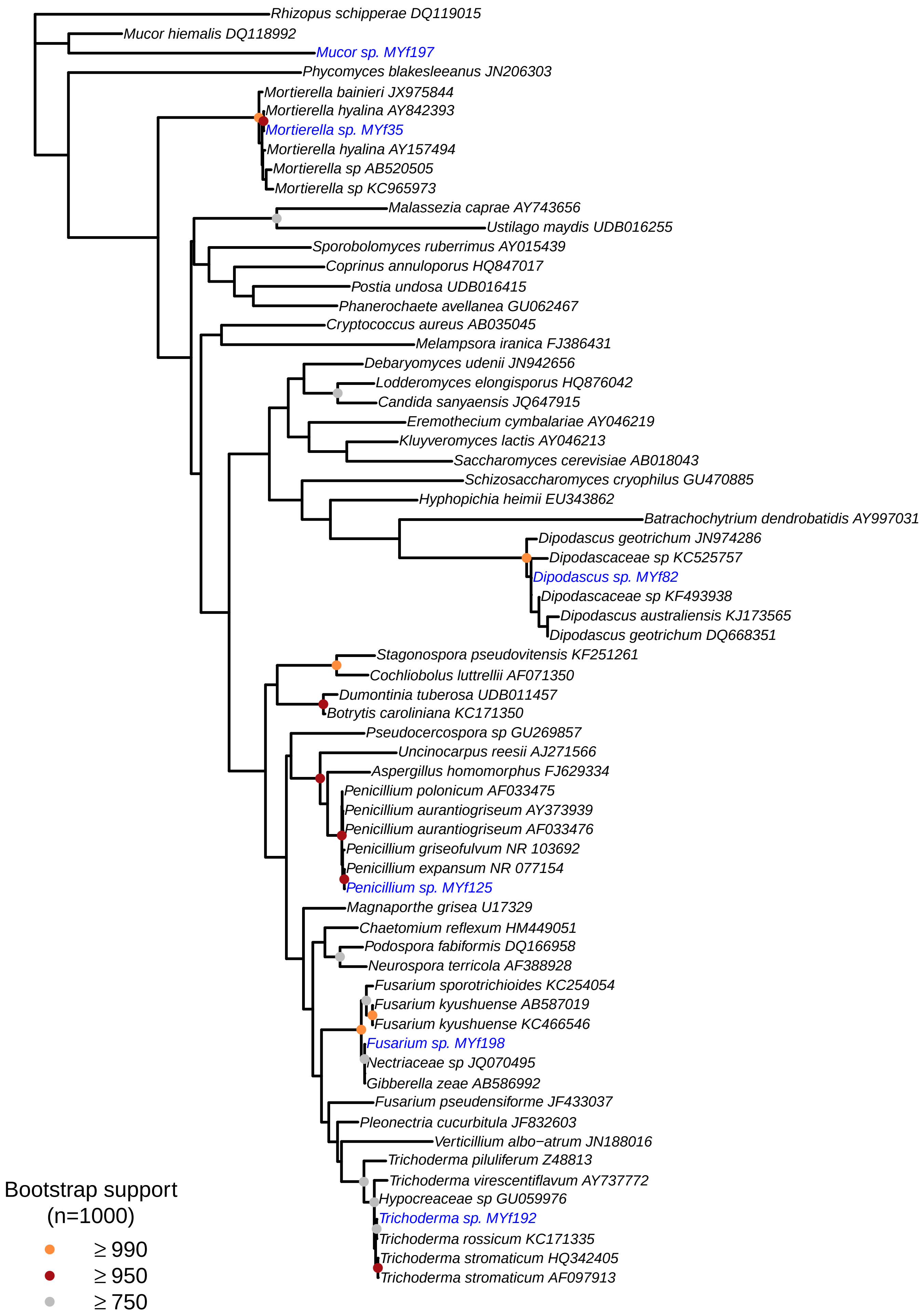
Rhizopus schipperae DQ119015
Mucor hiemalis DQ118992
Mucor sp. MYf197
Phycomyces blakesleeanus JN206303
Mortierella bainieri JX975844
Mortierella hyalina AY842393
Mortierella sp. MYf35
Mortierella hyalina AY157494
Mortierella sp AB520505
Mortierella sp KC965973
Malassezia caprae AY743656
Ustilago maydis UDB016255
Sporobolomyces ruberrimus AY015439
Coprinus annuloporus HQ847017
Postia undosa UDB016415
Phanerochaete avellanea GU062467
Cryptococcus aureus AB035045
Melampsora iranica FJ386431
Debaryomyces udenii JN942656
Lodderomyces elongisporus HQ876042
Candida sanyaensis JQ647915
Eremothecium cymbalariae AY046219
Kluyveromyces lactis AY046213
Saccharomyces cerevisiae AB018043
Schizosaccharomyces cryophilus GU470885
Hyphopichia heimii EU343862
Batrachochytrium dendrobatidis AY997031
Dipodascus geotrichum JN974286
Dipodascaceae sp KC525757
Dipodascus sp. MYf82
Dipodascaceae sp KF493938
Dipodascus australiensis KJ173565
Dipodascus geotrichum DQ668351
Stagonospora pseudovitensis KF251261
Cochliobolus luttrellii AF071350
Dumontinia tuberosa UDB011457
Botrytis caroliniana KC171350
Pseudocercospora sp GU269857
Uncinocarpus reesii AJ271566
Aspergillus homomorphus FJ629334
Penicillium polonicum AF033475
Penicillium aurantiogriseum AY373939
Penicillium aurantiogriseum AF033476
Penicillium griseofulvum NR 103692
Penicillium expansum NR 077154
Penicillium sp. MYf125
Magnaporthe grisea U17329
Chaetomium reflexum HM449051
Podospora fabiformis DQ166958
Neurospora terricola AF388928
Fusarium sporotrichioides KC254054
Fusarium kyushuense AB587019
Fusarium kyushuense KC466546
Fusarium sp. MYf198
Nectriaceae sp JQ070495
Gibberella zeae AB586992
Fusarium pseudensiforme JF433037
Pleonectria cucurbitula JF832603
Verticillium albo−atrum JN188016
Trichoderma piluliferum Z48813
Trichoderma virescentiflavum AY737772
Hypocreaceae sp GU059976
Trichoderma sp. MYf192
Trichoderma rossicum KC171335
Trichoderma stromaticum HQ342405
Trichoderma stromaticum AF097913
Bootstrap support
(n=1000)
≥ 990
≥ 950
≥ 750
